# Regulation of Notch signaling by non-muscle myosin II Zipper in *Drosophila*

**DOI:** 10.1007/s00018-024-05142-1

**Published:** 2024-04-24

**Authors:** Dipti Verma, Ankita Singh, Jyoti Singh, Mousumi Mutsuddi, Ashim Mukherjee

**Affiliations:** Department of Molecular and Human Genetics, Institute of Science, Banaras Hindu University, Varanasi, Uttar Pradesh 221005 India

**Keywords:** Endocytosis, Zipper, Notch signaling, Rab5, JNK pathway

## Abstract

The Notch pathway is an evolutionarily conserved signaling system that is intricately regulated at multiple levels and it influences different aspects of development. In an effort to identify novel components involved in Notch signaling and its regulation, we carried out protein interaction screens which identified non-muscle myosin II Zipper (Zip) as an interacting partner of Notch. Physical interaction between Notch and Zip was further validated by co-immunoprecipitation studies. Immunocytochemical analyses revealed that Notch and Zip co-localize within same cytoplasmic compartment. Different alleles of *zip* also showed strong genetic interactions with Notch pathway components. Downregulation of Zip resulted in wing phenotypes that were reminiscent of Notch loss-of-function phenotypes and a perturbed expression of Notch downstream targets, Cut and Deadpan. Further, synergistic interaction between Notch and Zip resulted in highly ectopic expression of these Notch targets. Activated Notch-induced tumorous phenotype of larval tissues was enhanced by over-expression of Zip. Notch-Zip synergy resulted in the activation of JNK pathway that consequently lead to MMP activation and proliferation. Taken together, our results suggest that Zip may play an important role in regulation of Notch signaling.

**Supplementary Information:**

The online version contains supplementary material available at 10.1007/s00018-024-05142-1.

## Introduction

Notch signaling is an evolutionarily conserved pathway that plays a fundamental role in various developmental events like cell fate determination, proliferation, apoptosis, and stem cell maintenance [1–4]. Notch is synthesized as a 300 kDa precursor protein that is subjected to its first cleavage in the trans-Golgi network by furin-like convertases (S1 cleavage), resulting in a 180 kDa N-terminal extracellular domain NECD (Notch extracellular domain) and a 120 kDa C-terminal transmembrane intracellular domain, N™ [5]. This heterodimer of Notch receptor is targeted to the cell membrane where it interacts with ligands of the DSL family (*Drosophila* Delta and Serrate (Jagged in mammals) and *C. elegans* LAG-2). Binding of ligands expressed on an adjacent cell to NECD leads to second proteolytic cleavage (S2) by ADAM family of metalloproteases in the extracellular portion of the NTM [6]. Ligand endocytosis is thought to generate mechanical force to induce a conformational change in the bound Notch receptor. This conformational change promotes the second cleavage of the receptor by metalloprotease [7, 8]. The second cleavage creates a membrane-tethered intermediate form of Notch, the NEXT (Notch extracellular truncation). This is followed by an intramembranous cleavage (S3) by γ-secretase complex (Presenilin, Nicastrin, PEN-2, and APH-1) and results in the release of Notch intracellular domain (NICD) from the membrane [9, 10]. The NICD then translocates to the nucleus with the aid of Importin α3 [11], where it transduces Notch signals by regulating the transcription of downstream target genes. In nucleus, NICD associates with the DNA binding protein CSL (mammalian CBF1/*Drosophila* Su(H)/*C. elegans* Lag-1) and facilitates the displacement of transcriptional co-repressors. The NICD–CSL complex then recruits Mastermind and other transcriptional coactivators leading to activation of Notch target genes such as the *Enhancer of Split* [*E*(*spl*)] complex genes in *Drosophila* [12–15]. These bHLH transcription factors, in turn, repress *achaete–scute complex* (As-C) proneural genes.

The same pathway can be deployed in numerous cellular contexts to play varied and critical roles for the development of an organism. The versatility of this pathway to influence different aspects of development comes from its multiple levels of regulation. We carried out different protein interaction screens to identify novel components involved in Notch signaling and its regulation. Independent protein interaction screens identified Zipper (Zip), also known as non-muscle myosin II (NM II), as an interacting partner of Notch. Non-muscle myosin II (NM II) is a hexameric actin-binding protein that consists of two heavy chains, two essential light chains and two regulatory light chains. It has contractile properties and is regulated by the phosphorylation of its light and heavy chains. In *Drosophila*, heavy chain of NM II is encoded by the *zipper*, and regulatory light chain is encoded by *spaghetti squash* [16, 17]. Zip plays a major role in regulating cell adhesion, cell migration, and determination of the polarity of migrating cells [18]. It has also been reported that Zip plays a crucial role in axon patterning, head involution and dorsal closure during embryonic development [19]. In the context of Notch signaling, it has already been shown that mechanical force has considerable implication in the activation of Notch receptor [20–22]. Recent studies have demonstrated that non-muscle myosin II-dependent tension across the Notch–Delta complex contributes to Notch activation [23]. Actomyosin contractility in cells having extensive lateral contacts with other cells has been shown to promote Notch activation and it was postulated that endocytosis and myosin-dependent pulling may both contribute to force-dependent Notch activation in these cells [23]. Further, presence of non-muscle myosin II at the nuclear periphery and its co-localization with the linker of nucleoskeleton and cytoskeleton (LINC) protein Nesprin2 and apical actin caps suggests that it plays role in transmission of cytoplasmic signals to the nucleus. Earlier it was reported that NM IIs can act as a mechano-transducer at the perinuclear area supporting the notion of actomyosin-mediated gene regulation through LINC [23].

In the present study, we have identified Zip as a Notch interactor using two independent protein–protein interaction screens and characterized the functional significance of this interaction. Using genetic and molecular studies, we have shown that Zip positively regulates Notch. Our co-immunoprecipitation experiments reconfirmed their physical interaction. *Zip* also genetically interacted with the components of Notch pathway. Further, loss-of-function of *zip* resulted in wing phenotypes identical to *Notch* loss-of-function wing phenotypes and compromised signaling, validating the integration of Zip with Notch signaling. *Notch* loss-of-function wing phenotype was rescued by over-expressing *zip* in the background. We have also shown the synergistic interaction between Notch and Zip. Additionally, increased levels of Notch targets, Cut, and Dpn upon *zip* co-expression with *Notch* clearly indicated the positive regulation of Notch by Zip.

## Materials and methods

### Yeast two-hybrid

A 393 bp *Drosophila* Notch cDNA (accession number M11664) fragment which encodes amino acids 1765–1895 containing NLS was amplified by polymerase chain reaction (PCR) and cloned in frame with the sequence encoding the LexA DNA-binding domain of bait vector. This construct was used as bait to screen oligo(dT)-primed *D*. *melanogaster* 0–24 h embryo cDNA libraries cloned in pGAD prey vectors containing GAL4 activation domains. A yeast two-hybrid screen was carried out as described previously [24]. Sequencing was performed for all three positive pGAD plasmids from *His* + colonies.

### Protein extraction, immunoprecipitation, immunoblotting, and mass spectrometry

Protein lysate was made using 1X RIPA lysis buffer (50 mM Tris–HCl pH 7.4, 1% NP-40, 0.25% sodium deoxycholate, 150 mM NaCl, 1 mM EDTA). The lysate containing 3 mg of protein was incubated with 5 μl antibody (anti-Notch antibody (C17.9C6), Developmental Studies Hybridoma Bank). 30 μl of A/G agarose beads was added to the lysate and it was rotated for overnight at 4 °C. After washing three times with 1X RIPA buffer, the samples were denatured and run at 12% SDS-PAGE gel to separate the peptides. This was followed by overnight transfer of proteins from gel onto a PVDF membrane. After blocking with 4% skimmed milk in TBST, the membrane was incubated with primary antibody followed by AP-conjugated secondary antibody. Chromogenic detection of signal was performed using Sigma FAST NBT/BCIP. 5.0 μl of anti-Notch antibody (C17.9C6) (Developmental Studies Hybridoma Bank) was used for immunoprecipitation. The primary antibodies used for western blotting were rabbit anti-Zip (1:1500) (kindly provided by Prof. Daniel P. Kiehart, Department of Biology, Duke University, Durham, NC) and rabbit anti-GFP (1:2000, Invitrogen).

For mass spectrometry, the separated protein samples in gel were stained with Coomassie Brilliant Blue. The sample preparation for mass spectrometry analysis was done as per the protocol of Bruker Daltonics adapted from Shevchenko et al., 1996 [25]. At first, the gel was excised into pieces with the help of scalpel and treated with washing solution (1:1 ratio of acetonitrile and 100 mM NH_4_HCO_3_) for 30 min. This step was accompanied with occasional vortexing at 300–350 RPM in order to destain the gel pieces. The gel pieces were then dehydrated by treating it with 100% acetonitrile for 3 min, followed by air drying. Dehydration was followed by reduction reaction where the gel pieces were treated with 10 mM dithiothreitol (DTT) dissolved in 100 mM NH_4_HCO_3_ for 30 min at 50 °C. Alkylation reaction was performed after this by treating the sample with 50 mM iodoacetamide (IAA) in100mM NH_4_HCO_3_ for 30 min in the dark at room temperature. The alkylated gel pieces were washed with a washing solution (1:1 ratio of acetonitrile and 100 mM NH_4_HCO_3_), and were dehydrated using 100% acetonitrile and subsequently air-dried. Overnight digestion was set up with 20 μl of 25 μg/ml proteomics grade trypsin solution (Promega Gold, USA) at 37 °C in a water bath. 0.1% trifluoroacetic acid (TFA) in 50% acetonitrile was used to extract the digested peptides from the gel pieces by vortexing for 10 min. This extraction step was repeated twice. The extracted peptides were concentrated using Speed-Vac and subjected to mass spectrometric analysis using AUTOFLEX speed MALDI-TOF/TOF instrument (Bruker Daltonics, Bremen, Germany).

### Proteomic analysis

Identification of the interacting partners of Zip was done by co-immunoprecipitation of the interacting proteins from GFP-Zip over-expression protein lysate with anti-GFP antibody followed by in-solution trypsin digestion and analysis by high-resolution mass spectrometry. The peptides identified in the HRMS screening were sorted on the basis of the sequence score (> 40). STRING database (https://string-db.org/) was used to generate the protein interactome based on identification of Zip interacting partners. Functional enrichment of the biological processes in the identified network represented as log 10 having *p* values with Benjamini–Hochberg correction has been shown in the bar graph. High-Resolution Accurate Mass Spectrometry System (Orbitrap Eclipse Tribrid Mass Spectrometer) from Thermo Fischer Scientific was used for the MS screening of Zip interacting proteins.

### *Drosophila* genetics

All fly stocks were maintained on standard cornmeal/yeast/molasses/agar medium at 25 °C as per standard procedures. Oregon-R flies were used as wild-type controls. *UAS-GFP-zip, UAS-GFP-Zip DN* (*UAS-Myo II-Neck-Rod*), *UAS-Myo II-Rod*, and *UAS-Myo II-Rod *(*delta N*_*term*_*58*) [26] were obtained as a gift from Prof. Daniel P. Kiehart (Department of Biology, Duke University, Durham, NC). *UAS-Notch-FL* [27]*, UAS-Notch-ICD, UAS-Notch-DN* [28], and Notch pathway components were kindly provided by Prof. S. Artavanis-Tsakonas (Department of Cell Biology, Harvard Medical School, Boston, MA).

- *cn*^*1*^* bw*^*1*^* sp*^*1*^* zip*^*1*^*/CyO* (BDSC 4199),
- *P*{*FRT*(*w*^*hs*^)}*G13 zip*^*2*^*/CyO* (BDSC 8739)*,*
- *P*{*lacZ.w*^+^}*276*, *y*^*1*^ sc* v^*1*^* sev*^*21*^*; P*{*TRiP.GL00623*}*attP40* (BDSC 37480), *y*^*1*^ sc* v^*1*^* sev*^*21*^*; P*{*TRiP.HMS01618*}*attP2* (BDSC 65947)*,*
- *Sqh*^*AX3*^*-GFP* (BDSC 57144)*, UAS-Bsk-DN* (BDSC 6409)*, vg-GAL4* (BDSC 8222)*, GMR-GAL4* (BDSC 8121)*, ptc-GAL4* (BDSC 2017)*, en-GAL4* (BDSC 30564), *ap-GAL4* (BDSC 56807), *dpp-GAL4* (BDSC 1553), and *C96-GAL4* (BDSC 43343) stocks were obtained from Bloomington stock center. All crosses were performed at 25 °C. The combination lines *vg-GAL4/UAS-GFP-zip* and *UAS-Notch-DN/C96-GAL4* were made with the help of appropriate genetic crosses.

### Wing mounting

For the preparation of wings from adult flies, F1 progeny flies were taken and their wings were separated with the help of needle and scalpel. The wings were then transferred on a glass slide and washed with isopropyl alcohol. This was followed by mounting the wings immediately in 70% glycerol. Wing images were taken using Brightfield Nikon Eclipse Ni microscope.

### Immunocytochemistry and confocal microscopy

*Drosophila* third instar larvae were dissected in chilled 1X PBS and fixed in 4% Paraformaldehyde for 20 min. The tissues were then washed with washing solution (0.1% BSA in Tri-PBS) for 4 times, 15 min each. This was followed by blocking the tissues using blocking solution (PBST with 8% of serum) for 30 min to an hour. The tissues were then incubated with primary antibody for overnight at 4 °C. On the next day, the tissues were washed again for 4 times, 20 min each followed by blocking for 30 min to 1 h at room temperature. This was followed by incubating the tissues with fluorophore-conjugated secondary antibody for 90 min at room temperature. Tissues were then washed again for 4 times, 20 min each followed by a wash in 1X PBS. DAPI was added in the dark for 20–30 min. The tissues were washed again with washing solution for 4 times and once with 1X PBS for 15 min each. The discs were finally dissected in chilled 1X PBS and incubated in DABCO for overnight. Next day, the tissues were mounted and observed under Carl Zeiss LSM 780 laser scanning confocal microscope. The images were processed using Adobe Photoshop 7. The primary antibodies used in this study are as follows: mouse anti-Notch (C17.9C6; 1:300), mouse anti-Notch (C458.2H; 1:100), mouse anti-Cut (2B10; 1:100), mouse anti-MMP1 (3A6B4; 1:100), mouse anti-Delta (1:100), Guinea Pig anti-Rab5 (1:1000, a generous gift from Prof. Akira Nakamura, Institute of Molecular Embryology and Genetics, Kumamoto, Japan), mouse anti-Armadillo (N2 7A1; 1:100) (all from Developmental Studies Hybridoma Bank), rabbit anti-Cleaved Caspase 3 (1:50), rabbit phospho-JNK (1:100), rabbit anti-phospho myosin regulatory light chain 2 (1:50) (Cell Signaling Technology), rabbit anti-PH3 (1:50) (Merck), rabbit anti-Dpn (1:100) (kindly provided by Prof. Yuh N Jan, Howard Hughes Medical Institute, University of California San Francisco, California), rabbit anti-Zip (1:200). Phalloidin stain was used to detect actin. Alexa488-, and Alexa555-conjugated secondary antibodies (1:200, Molecular Probes) were used to detect the primary antibodies.

### Statistical analysis

Intensity profiling in the *Drosophila* imaginal discs and quantification of the area covered by tumorigenic discs representing the size of the discs was done using Image J. 5–10 imaginal discs were used for the quantification purpose in each case. Integrated density/area of the domain indicated the intensity of the staining in confocal images. Image J was also used to quantify the number of PH3 positive cells in the wing imaginal discs where all the cells were counted using analyze particles feature in the software. The RGB plot profile feature of Image J was also used to denote the intensity of Notch targets in Zip compromised condition compared to the internal control. The line icon was used to measure the area of the pouch region of the wing discs with Cut and Dpn expression. RGB plot profile feature from plugins section was then used to plot intensity graphs. The error bars in the graphs denoted the standard error of the mean value from the replicated experiments. One-way analysis of variance (ANOVA) followed by Tukey’s multiple comparison test and unpaired t test was used to determine the extent of significance among different genotypes. *p* value < 0.05 was accepted as statistically significant. GraphPad Prism 5 was used for plotting graphs.

## Results

### Zip is an interacting partner of Notch

In an effort to identify novel components involved in Notch signaling and its regulation, we carried out two independent protein interaction screens, one based on the identification of cellular protein complexes using immunoprecipitation followed by mass spectrometry and other based on yeast two-hybrid system. Both the screens identified Zip as an interacting partner of Notch. For immunoprecipitation, tissue lysate was prepared from hyperplastic wing imaginal discs over-expressing *Notch-ICD* under *vg-GAL4* driver and from wild type wing discs as well that served as the control. Anti-Notch antibody was used to precipitate Notch along with its interactors from both the lysates. The protein bands exclusively present in the lane with over-expressed Notch lysate and absent in the control lane were excised from the SDS-PAGE and were processed for mass spectrometry. One of them was identified as Zip, a 205 kDa protein as a novel interactor of Notch (Fig. S1). Zip was also recovered in a standard yeast two-hybrid screen in which approximately 6 × 10^6^ cDNAs from a random primed *Drosophila* 0–24 h embryonic library were screened with a cDNA corresponding to amino acids 1765–1895 of *Drosophila* Notch fused in frame to the LexA DNA binding domain used as a bait [24]. In the same screen, multiple positive clones of Su(H), a well-established binding partner of Notch-ICD, were also identified, which validated our approach. Sequence analysis of three identified positive clones of Zipper (Zip) revealed that the carboxy-terminal part of Zip (amino acids 1692–1972) binds to Notch-ICD. In addition, we have also carried out a large-scale proteomic analysis of Zip based on co-immunoprecipitation of Zip along with its interacting proteins followed by mass spectrometry (MS). Notch was identified as one of the many interacting partners in our MS analysis. Along with Notch, many other cytoskeletal components and endocytic machinery proteins were also identified in the MS analysis (Fig. S7).

**Fig. 1:**
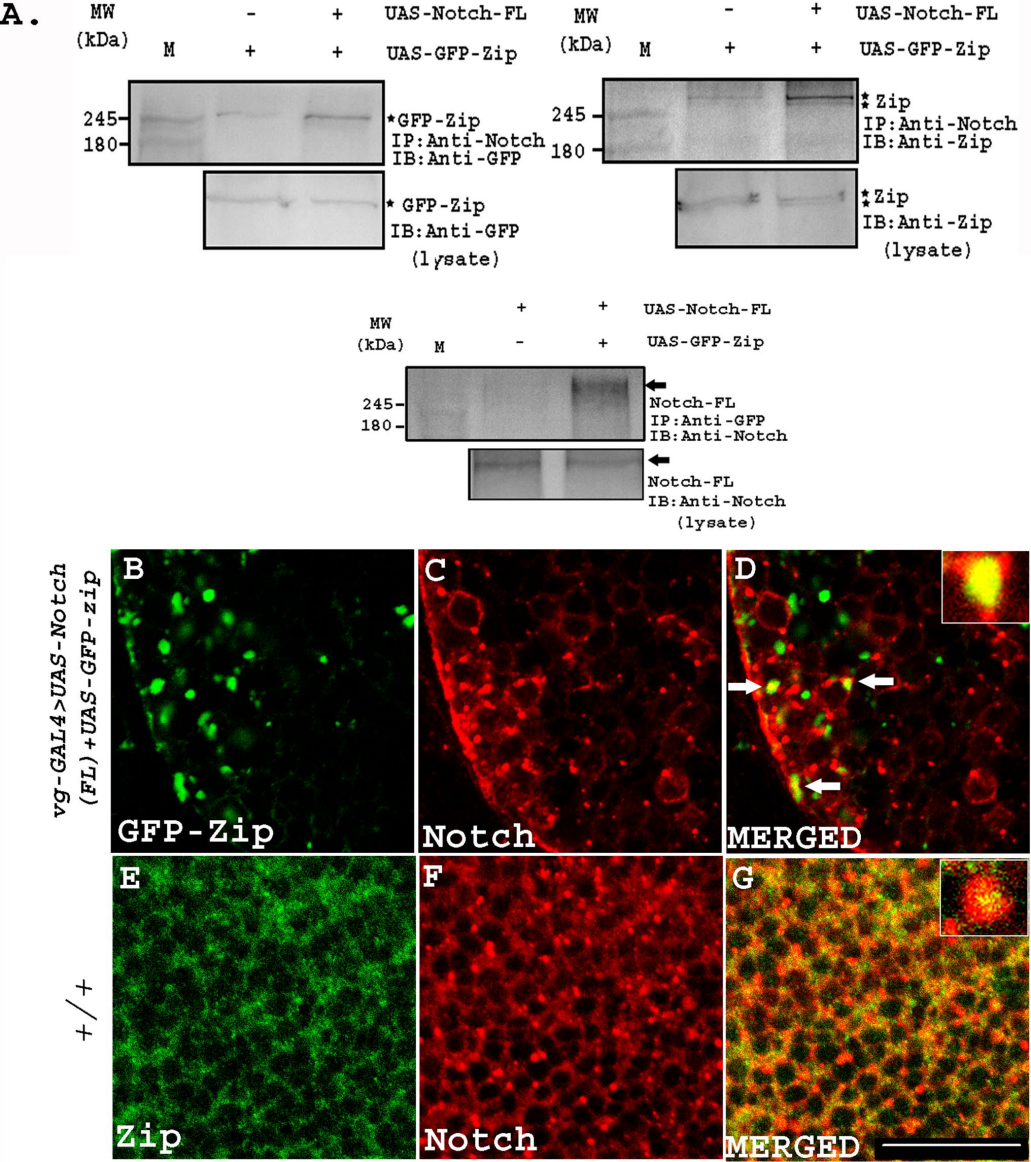
**Physical interaction between Notch and Zip**. **A** Zip was identified as an interacting partner of Notch. Co-immunoprecipitation was carried out with head tissue lysates over-expressing GFP-Zip and Notch FL. *M* indicates the marker lane, (+) symbol indicates the presence and (−) shows the absence of the specified reagent. Notch-FL and endogenous Notch immunoprecipitated endogenous and GFP-tagged-Zip that was detected by anti-GFP and anti-Zip antibody on the western blot. Star marks indicate the bands of GFP-tagged Zip and endogenous Zip. No GFP-Zip protein bands were observed in the negative control (Fig. S1C). In the other direction, anti-GFP immunoprecipitated Notch-FL that was detected by anti-Notch antibody on the western blot. Lower blots show the presence of the specified protein bands in the experimental and the control lysates. **B**–**D** **Zip colocalizes with Notch in the over-expressed condition in the cytoplasmic compartment.** Zip and Notch-FL were co-expressed under *vg-GAL4*. GFP-tagged Zip forms cytoplasmic aggregates upon over-expression and colocalizes with Notch-FL puncta on the cytoplasmic membrane. Anti-Notch antibody was used for the detection of over-expressed full-length Notch. Panel **D** is the merged image of **B**, **C**. The insat image in the panel D represents enlarged view of the image showing extent of colocalization between Notch and Zip. **E**–**G** Zip colocalizes with Notch in wild type wing discs. Merged image of panel G shows that Zip colocalizes with Notch on the cytoplasmic membrane. The antibody used for detection of wild type Zip in panel **E** is anti-Zip and for Notch detection in panel **F** is anti-Notch. The insat image in the panel G represents enlarged view of the images showing extent of colocalization between these two proteins. Scale bar: 20 μm

Physical interaction of Notch with Zip was further validated by co-immunoprecipitation studies. Protein lysate was prepared from adult head tissues co-expressing full-length Notch (Notch-FL) and GFP-Zip driven by *GMR-GAL4*. Anti-Notch antibody was used to immunoprecipitate Notch along with its interacting partners in a complex which were fractionated on SDS-PAGE. Co-immunoprecipitated GFP-conjugated Zip protein was detected on a western blot using anti-GFP antibody (Fig. 1A). In addition, we also used anti-Zip antibody that detected both endogenous and over-expressed GFP-Zip that appeared as doublet band on the western blot (Fig. 1A). In the same set of experiments, we also demonstrated the physical interaction of over-expressed Zip with endogenous Notch protein. As in case of over-expressed Notch, Zip was also detected as a physically associating partner of the endogenous Notch with anti-GFP as well as anti-Zip antibody (Fig. 1A). To rule out the presence of Zip in our western blot due to non-specific binding with AG beads, we also performed immunoprecipitation experiment without adding primary antibody. No expression of Zip could be seen in the negative control compared to the presence of a doublet band of endogenous and over-expressed GFP-tagged Zip in the experimental lanes (Fig. S1C). Conversely, we used anti-GFP antibody to immunoprecipitate GFP-tagged Zip along with its binding partners and Notch FL was found to be co-immunoprecipitated with Zip which was detected using anti-Notch antibody on the western blot (Fig. 1A). In the same set-up, we also performed co-immunoprecipitation studies using protein lysate from only Notch FL over-expressed condition. Here, anti-GFP antibody could not immunoprecipitate endogenous Zip (absence of over-expressed GFP-Zip). Hence, we did not observe presence of Notch upon immunoblotting with anti-Notch antibody (Fig. 1A). These results confirmed that Notch is indeed physically associated with Zip.

To corroborate our physical interaction experiments of Notch and Zip, we performed colocalization experiments through immunostaining using anti-Notch and anti-Zip antibodies. We checked their localization in GFP-tagged Zip and Notch-FL co-expressed larval wing imaginal discs. We observed that Zip colocalized with Notch in over-expressed condition on the cell membrane and in the cytoplasm (Fig. 1B–D, Fig. S1G–G″). As in case of colocalization of Notch and Zip in over-expressed condition, endogenous Zip and Notch also colocalized in the same cytoplasmic compartment in the wild-type wing imaginal disc (Fig. 1E–G). In the over-expressed condition, GFP-tagged Zip was found to form large cytoplasmic aggregates [29–31], and these aggregates colocalized with Notch-FL (Fig. 1B, D) indicating that the two proteins colocalize and perhaps functionally regulate developmental processes together.

### *z*ip genetically interacts with Notch pathway components

**Fig. 2:**
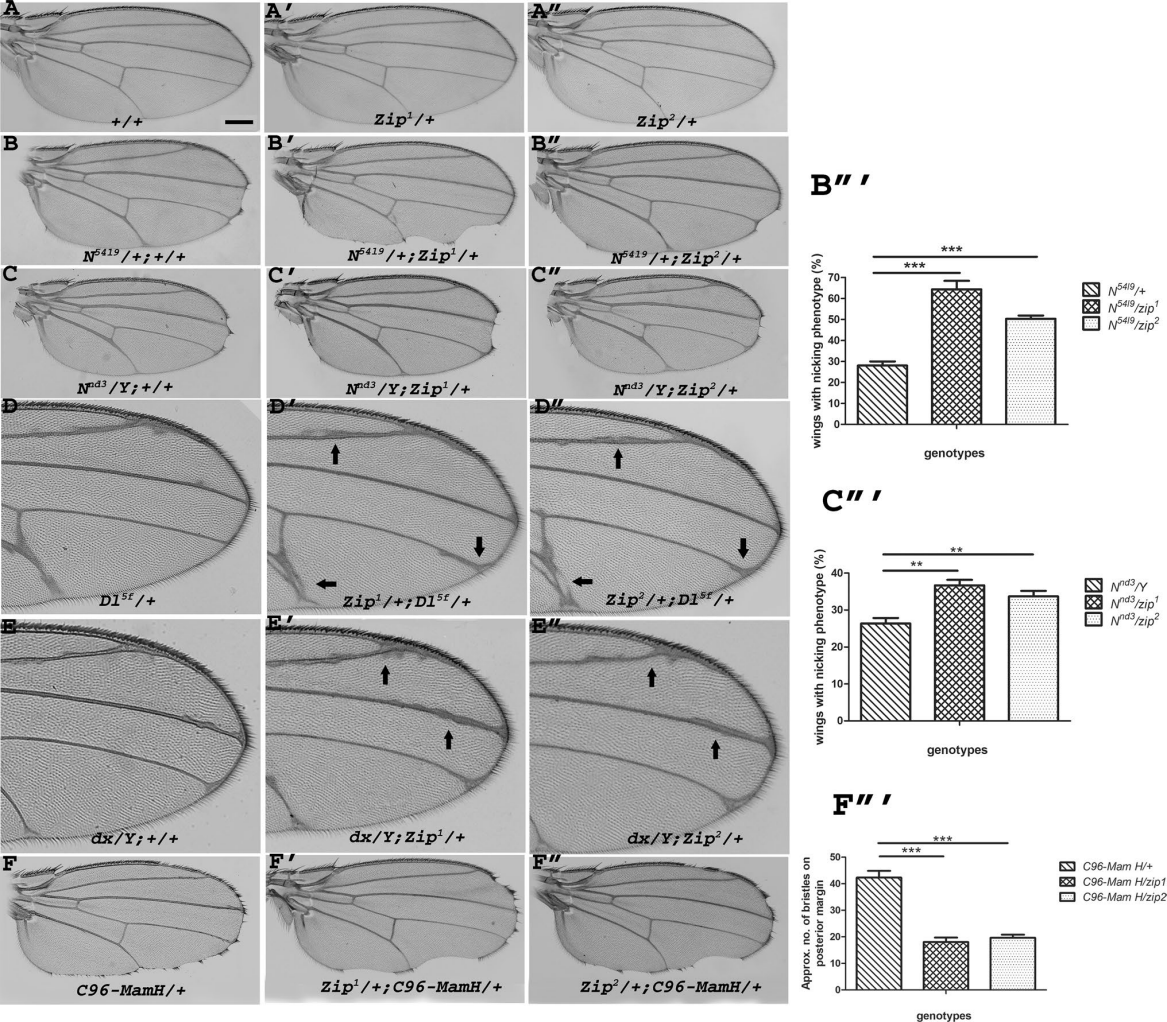
Genetic interaction of *zip* with Notch pathway components. Representative wings from different Notch pathway component mutants are shown in first column and in trans-heterozygous condition with *zip* mutants *zip*^*1*^ and *zip*^*2*^, are shown in second and third column respectively. Wing from wild type is shown in **A** and from *zip* alleles are shown in **A**′–**A**″. **B**-**C**″ Wings from heterozygote *N*^*54l9*^ (**B**) and hemizygous *N*^*nd−3*^ (**C**) shows nicking phenotype which were further increased in number in trans-heterozygous combination with loss-of-function alleles of *zip*, *zip*^*1*^ (**B**′, **C**′), and *zip*^*2*^ (**B**″, **C**″). **D**–**E**″ Heterozygous wings of *DL*^*5f*^ (**D**) and hemizygous *dx* (**E**) showed vein thickening phenotype which was enhanced in trans-heterozygous condition with *zip* mutants (**D**′, **D**″ and **E**′, **E**″) respectively. (**F**–**F**″) Representative wings of loss-of-function allele of *Mastermind* driven by *C96-GAL4* showed serrated wing margin phenotype, which was further enhanced by combining *zip* alleles trans-heterozygously. (**B**‴, **C**‴ and **F**‴) Graphs representing the percentage of wings showing nicking phenotype (**B**‴ and **C**‴) and approximate number of bristles on the posterior margin of the wing (**F**‴) in trans-heterozygous combination of *zip* mutants with the components of Notch signaling. (**B**‴ and **C**‴) All experiments were performed in triplicates (*n*). For consistency a total of 80 flies/vial was observed (**B**‴) No. of wings with nicking phenotype *N*^*54l9*^/ + : n1 = 22/80, n2 = 20/80 and n3 = 24/80; *N*^*54l9*^/*zip*^*1*^: n1 = 48/80, n2 = 42/80 and n 3 = 52/80; *N*^*54l9*^/*zip*^*2*^: 42/80, n2 = 40/80 and n3 = 46/80. (**C**‴) No. of wings with nicking phenotype *N*^*nd3*^: n1 = 22/80, n2 = 22/80 and n3 = 24/80; *N*^*nd3*^/*zip*^*1*^: n1 = 30/80, n2 = 27/80 and n3 = 32/80; *N*^*nd3*^/*zip*^*2*^: 28/80, n2 = 20/80 and n3 = 36/80. **F**‴ The genotype of the flies mentioned on the *X*-axis of the graph are as follows: *C96-GAL4/UAS-Mam H, C96-GAL4/UAS-Mam H* + *zip*^*1*^*, C96-GAL4/UAS-Mam H* + *zip*^*2*^. Unpaired *t*-test was performed to determine *p*-value (**p < 0.01, ***p > 0.001). Scale bar: 3 cm

To address the functional implications of physical interaction between Zip and Notch, we studied the genetic interactions between *zip* mutants and mutants of Notch pathway components in trans-heterozygous combinations. We used two independent amorphic alleles of *zip*, *zip*^*1*^, and *zip*^*2*^. We checked their genetic interaction with the Notch mutants, the amorphic allele of *Notch*, *N*^*54l9*^, and the hypomorphic allele of Notch, *N*^*nd*3^. A trans-heterozygous combination of *zip* alleles with *N*^*54l9*^ and *N*^*nd*3^ resulted in an increased number of flies with wing-nicking phenotype (Fig. 2B–C″). It was observed that 28% of *N*^*54l9*^ mutant flies exhibited wing-nicking phenotype which was increased to 60% and 52% (42/80) when these flies were combined heterozygously with *zip*^*1*^ and *zip*^*2*^ alleles, respectively (Fig. 2B‴). Similarly, 28% of *N*^*nd*3^ mutant flies exhibited wing-nicking phenotype which was increased to 37% and 35% when these flies were combined heterozygously with *zip*^*1*^ and *zip*^*2*^ alleles respectively (Fig. 2C‴). This indicated a further reduction of Notch signaling upon lowering the dose of *zip* in the same background thus validating a functional relevance between the two genes. The wing vein thickening phenotype of Delta (*Dl*^*5f*^) was also enhanced by reducing the dose of *zip* (Fig. 2D–D″). The null allele of *deltex* (*dx*), a cytoplasmic modulator of Notch resulted in mild wing-vein thickening. However, we noticed an enhancement in wing vein thickening phenotype in *dx* hemizygous condition with *zip* alleles. Since vein thickening is a *Notch* loss-of-function phenotype, the enhancement of it with *zip* alleles in the background indicates further decrease of Notch signaling upon lowering the dose of Zip (Fig. 2E–E″). The functional interaction predicted to be caused by the colocalization of Notch and Zip in the same cytoplasmic compartment (as shown in Fig. 1B–G) is evident from genetic interaction between *zip* and *Notch* mutant alleles (Fig. 2B–C‴). Additionally, we also checked the genetic interaction with *C96-GAL4*-driven dominant-negative form of C-terminal Mastermind truncation (MamH), that display a fully penetrant wing-nicking phenotype [32, 33]. Reducing the dose of *zip* in this background elicited enhanced wing notching combined with reduced marginal bristles. It was observed that the approximate number of bristles exhibited by *C96-Mam H* flies was 40 on the entire posterior margin which was reduced to 19 and 21 when the flies were heterozygously combined with *zip*^*1*^ and *zip*^*2*^ alleles (Fig. 2F‴). This modulation in the wing phenotype could be attributed to a compromised dose of Zip in the cytoplasm that ultimately affects the downstream Notch signaling processes, resulting in an enhanced wing-nicking and loss of marginal bristles in the background of *C96-GAL4*-driven dominant-negative Mastermind (Fig. 2F–F″). The genetic interaction between *zip* and *Notch* pathway components demonstrated that effects caused by decreased Notch signaling were further enhanced when *zip* mutants were brought in trans-heterozygous combination indicating that Zip positively regulates Notch signaling and its loss results in the reduction of Notch signaling (Fig. 2).

### The loss-of-function of *zip* renders wing phenotypes similar to Notch mutant phenotypes and perturbs the expression pattern of Notch targets, Cut and Deadpan

The integration of Zip with Notch signaling was further verified by loss-of-function studies in which Zip was downregulated using *UAS-zip-RNAi* and *UAS-GFP-zip DN* under various wing-specific GAL4 driver lines. *vg-GAL4*-driven *UAS-zip-RNAi* displayed slightly bent, crumpled wing phenotype with serration on the lower margin of the wing blade. Some of these wings also exhibited irregular marginal bristles with thickened veins and extra vein material. Similarly, abrogating Zip using *UAS-GFP-zip DN* in the ventral domain of the wings using the same *vg-GAL4* driver resulted in wing-nicking phenotype (Fig. S2A–A′). Down-regulating Zip using *UAS-zip-RNAi* in the dorsal region of the wing with *ap-GAL4* yielded outward-directed erect wings with a crumpling phenotype. These wings also harbored several other phenotypes including irregular marginal bristles, extra vein material, and extra cross-veins. Similarly, eliminating Zip using *UAS-GFP-zip DN* in the dorsal region of the wing resulted in pupal lethality (Fig. S2B–B″). *en-GAL4-*driven *UAS-zip-RNAi* resulted in 100% pupal lethality. However, blocking Zip using Zip-DN in this region with same *Gal4* driver resulted in wings harboring various phenotypes similar to Notch loss-of-function that included wing-nicking, mispatterning of wing hair, disruption of vein pattern and the cross-veins, and wing blister (Fig. S2C–C″). *C96-GAL4*-driven *UAS-zip-RNAi* resulted in slightly bent wings with fully penetrant serration on the lower margin of the wing blade. These wings also exhibited mild crumpling with ectopic bristles on the anterior portion of the wings. Reducing the activity of Zip using *UAS-GFP-zip DN* in the wing margin resulted in wing-nicking phenotype (Fig. S2D–D″). Reducing the dose of *zip* on the anterior/posterior boundary using *ptc-GAL4* resulted in pupal lethality with very small number of flies eclosing that harbored wing-nicking on the anterior/posterior region of the wing blade (Fig. S2E–E″). Down-regulating *zip* at the anterior/posterior boundary using *dpp-GAL4* displayed disrupted wing bristles and mild wing-nicking at A/P boundary (Fig. S2F–F″). These phenotypes shown by downregulation of *zip* using different *GAL4* lines are reminiscent to *Notch* loss-of-function phenotypes, indicating that lowering the dose of Zip results in further reduction of the Notch signaling (Fig. S2G).

**Fig. 3:**
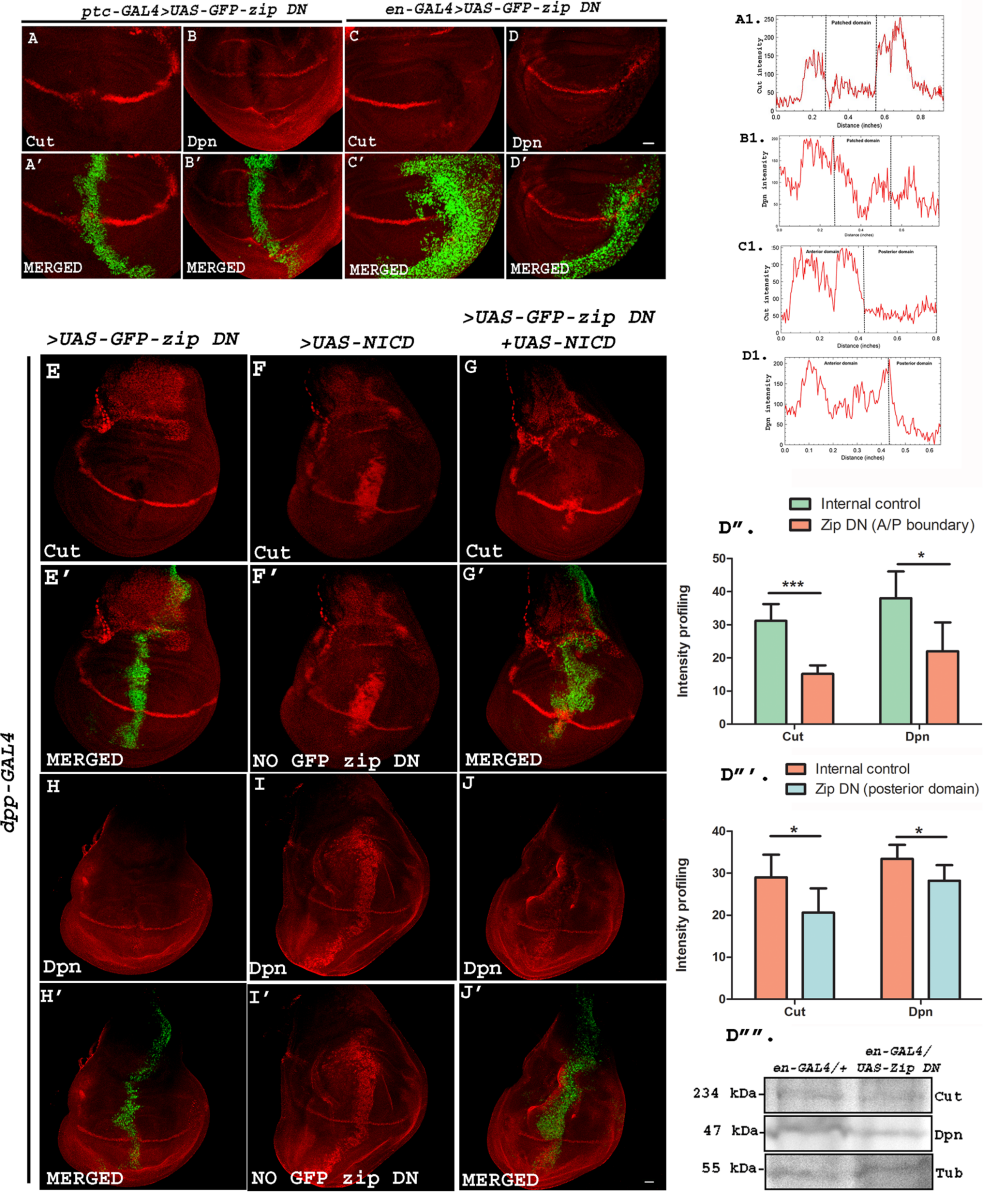
**A**–**D** Reducing the dose of Zip results in lowered Notch signaling. Representative wing discs display the expression pattern of Cut and Dpn upon eliminating Zip on the A/P boundary and in the posterior region using *ptc-GAL4* and *en-GAL4* respectively. Abrogating Zip in the *patched* and *engrailed* domain using *UAS-GFP-zip DN* results in the perturbed expression of Cut (**A**, **C**) and Dpn (**B**, **D**) in these regions. **A**′–**D**′ Merged images showing the expression pattern of Cut and Dpn along with the *patched* and *engrailed* domain marked by GFP-tagged Zip DN. **A1**–**D1** RGB plot profiles indicating the intensity of Cut and Dpn in **A**, **B**, **C** and **D**, respectively. **E**–**J**′ Loss of Cut and Dpn expression due to Zip DN on the A/P boundary using *dpp GAL4* (**E** and **H** respectively) is rescued by over-expressing Notch ICD in the background (**G** and **J** respectively). Over-expressing *UAS-Notch-ICD* with *dpp-GAL4* resulted in ectopic expression of Cut and Dpn throughout A/P boundary (**F** and **I** respectively). (**E**′–**G**′ and **H**′–**J**′) Merged images showing the expression of Cut and Dpn in dpp domain with GFP-tagged *UAS-zip-DN* (**E**′ and **H**′), and GFP-tagged *UAS-zip-DN* + *UAS-Notch ICD* (**G**′ and **J**′). (**F**′ and **I**′) Merged images showing the expression of Cut and Dpn along AP boundary with over-expressed Notch ICD. Absence of GFP indicates the absence of *GFP-zip DN*. (**D**″–**D**‴) Graphs representing the intensity profiling of Cut and Dpn upon abrogation of Zip at the A/P boundary (**D**″) and in the posterior region (**D**‴) of the wing imaginal discs. Unpaired *t*-test was performed to determine the *p*-value (*p < 0.05, ***p < 0.001). **D**″″ Western blot showing the expression of Cut, Dpn and internal control Tubulin (Tub). Scale bar: 20 μm

The modulation of the Notch signaling caused by down-regulation of Zip was further validated in the wing imaginal discs by examining the expression level of Notch downstream targets, Cut, and Dpn. Cut encodes a homeodomain transcription factor that bears a significant structural and functional similarity with several vertebrate proteins. Notch is required for the activation of Cut in a cell-autonomous manner [34]. Similarly, activated Notch directs the expression of Dpn by binding to the Notch-responsive enhancer present in the regulatory region of Dpn [35]. *ptc-GAL4* and *en-GAL4*-driven *UAS-GFP-zip DN* in third instar larvae resulted in the perturbation of the expression of Cut and Dpn*.* RGB Plot profiles display the decreased fluorescence intensity of Cut and Dpn in the regions with compromised Zip compared to the internal control (Fig. 3A1–D1). The expression of Cut and Dpn has been observed to be normal in the wing imaginal discs of wild type flies (Fig. S3A, B). These observations confirmed that Zip not only positively regulates Notch signaling but also play a vital role in the modulation of Notch downstream targets (Fig. 3). Perturbation of Notch targets, Cut, and Dpn upon down-regulating Zip at A/P boundary (using *ptc-GAL4*) and in posterior domain (using *en-GAL4*) was observed in all the discs that were examined (total number of wing discs examined = 30). Image J was used for the intensity profiling where integrated density/area of the domain was used for quantification purpose. A total number of 5 discs were used for quantification in each case which was subjected to unpaired t-test to determine the significance of our findings. Mean intensity for internal control of Cut was 29, whereas that of *en-GAL4* > *UAS-GFP-zip DN* was 20.6. Mean intensity for internal control of Dpn was 33.4, whereas that of *en-GAL4* > *UAS-GFP-zip DN* was 28.2. In case of *ptc GAL4*-driven *UAS-GFP-zip DN*, mean intensity of internal control for Cut expression was 31.2, whereas that of *Zip DN* domain was 15.2. For Dpn, mean intensity for internal control was 38, whereas *UAS-GFP-zip DN* was 22.

To verify whether the diminished expression of Cut and Dpn downstream to Zip-DN is mediated by reduced activity of Notch signaling, we supplied Notch-ICD in the Zip compromised background. Over-expression of *UAS-GFP-zip DN* using *dpp-GAL4* alone leads to Cut and Dpn reduction in the D/V boundary straddling the A/P domain of *dpp-GAL4* (Fig. 3E, H), whereas both Cut and Dpn were found to be ectopically increased upon over-expressing Notch ICD in the Dpp domain (Fig. 3F, I). When *UAS-Notch ICD* was over-expressed in the background with dominant-negative *zip,* the Notch targets were found to be rescued thus indicating that loss of Cut and Dpn resulting due to perturbed Zip is Notch-mediated (Fig. 3G, J), thus highlighting the role of Zip in modulating the activity of Notch signaling. To validate the loss of Notch signaling targets in Zip DN background, we prepared protein lysates from wing discs of *en-GAL4* > *Oregon R* and *en-GAL4* > *UAS-GFP-zip DN* and checked for the expression of Cut and Dpn using Western blotting. Both Notch downstream targets were found to be reduced in Zip DN condition compared to the control condition (Fig. 3D″″).

### The loss-of-function of *zip* leads to cell surface accumulation of Notch receptor and endocytic component Rab5

**Fig. 4:**
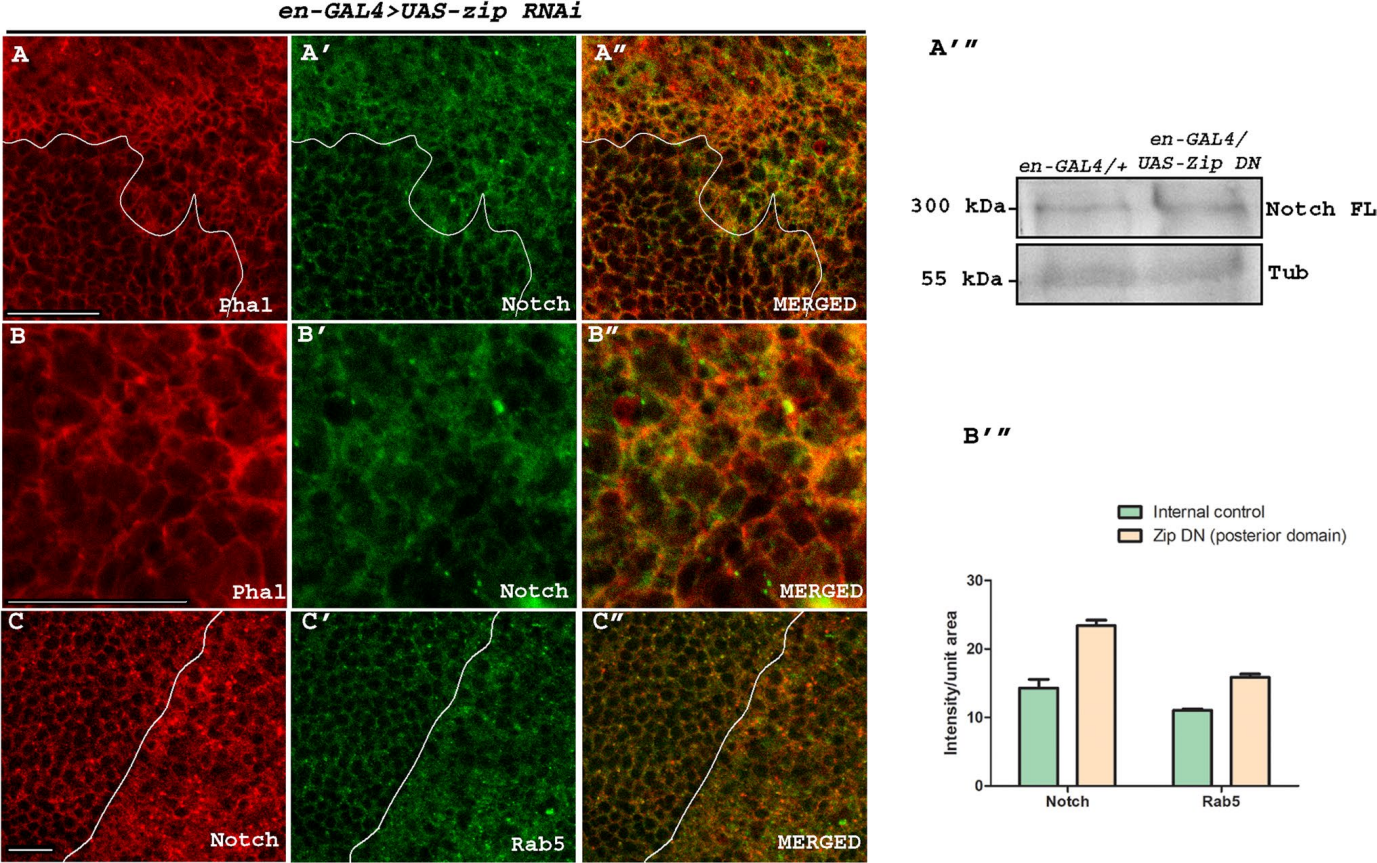
Loss of Zip leads to accumulation of Notch receptor and Rab5. **A**–**B**′″ Zip downregulation with *UAS-zip RNAi* in the posterior region of the wing disc using *en-GAL4* resulted in an accumulated expression of the Notch receptor (**A**′) and actin (**A**) on the cell membrane compared to its endogenous expression in the anterior domain (**A**). Panel **A**″ represents a merged image of **A** and **A**′ where Notch was observed to colocalize with Phal. Panel **B**–**B**′ shows the expression of Phal, Notch at a higher resolution. Panel **B**″ represents merged image of **B** and **B**′. (**A**′″) Western blotting showing the expression of Notch in control and Zip compromised condition. **C**–**C**″ Down-regulation of Zip using *UAS-zip RNAi* with *en-GAL4* resulted in accumulated Notch (**C**) and Rab5 (**C**′) in the posterior region of the wing disc. Panel **C**″ is the merged image of **C** and **C**′. Scale bar: 20 µm. **B**‴ Graph representing the accumulation of Notch and Rab 5 upon downregulating Zip in the posterior region. Error bar denotes the error between the mean values of the specified genotypes

Since down-regulation of Zip leads to reduced expression of Notch targets, we wanted to check the status of Notch receptor in Zip compromised background. Down-regulation of Zip in the posterior domain of the wing disc in *UAS-GFP-zip DN* using *en-GAL4* driver*,* resulted in altered Notch localization. The wild type membranous Notch appeared to be accumulated in Zip compromised domain. These studies indicated that loss of Zip might result in perturbation of Notch receptor processing subsequently leading to down-regulated Notch signaling (Fig. S4A–A″). An accumulation of Notch in Zip compromised domain of the wing discs was observed in 100% of the wing discs that were examined (total number of discs examined = 20). A quantification of the membranous Notch expression was used as a readout for accumulated Notch at the cell surface. About 10 discs were used for the quantification purpose using Image J. Mean intensity of the wild type expression of Notch in the internal control was 22, whereas that in the posterior domain with compromised Zip was 31.5. To verify the accumulation of Notch at the cell surface, we also checked the colocalization of accumulated Notch with membrane marker actin. Actin associates with the plasma membrane and provide mechanical support, determine cell shape, etc. [36]. Here, accumulated Notch in the posterior region with compromised Zip using *UAS-zip RNAi* with *en-GAL4* was observed to colocalize with Phalloidin marked actin (Fig. 4A–B″). Western blotting also showed a higher band intensity of Notch FL in the Zip compromised condition compared to the wild type that served as the control (Fig. 4A‴).

Accumulation of the Notch receptor at the cell surface has been correlated with impaired endocytosis of the receptor in the signal receiving cell leading to compromised signaling [37]. Myosin II is implicated in the endocytic process where via force generation, it assists in pulling the clathrin-coated pit along with the receptor toward inside of the cell leading to invagination of the membrane thus initiating the process of early endosome formation [38]. Rab5 belongs to the family of GTPases that regulate trafficking into and between the early endosomes [39]. Hence, to understand the mechanism behind accumulation of Notch receptor in the signal receiving cell upon abrogating Zip, we checked the status of early endosomal marker Rab5 in Zip compromised condition. Down-regulating Zip using *UAS-zip RNAi* with *en-GAL4* revealed an accumulated pattern of Notch at cell surface in the posterior domain of the wing imaginal disc. Similar to Notch accumulation, Rab5 also appeared to be accumulated in the engrailed domain compared to the anterior domain with endogenous Zip. Accumulated Notch was observed to partially colocalize with Rab5 (Fig. 4C–C″). This suggested that perturbation of Zip compromised the formation of early endosome as evident from the accumulated expression pattern of Rab5. Loss of Zip led to loss of pulling force required for the formation of endocytic vesicles indispensable for receptor internalization, leading to accumulated Notch receptor at the cell surface. Our findings suggested that Zip is necessary for Notch receptor internalization in the signal receiving cell leading to activation of the signaling pathway. The accumulation of Notch along with Rab5 was observed in all the wing discs that were examined (total number of wing discs examined = 15). Among them, 5 discs were used for the quantification of the intensity.

To rule out the involvement of the regulatory light chain in the functional role of Zipper in Notch regulation, we also examined the status of Notch in regulatory light chain compromised background. The gene *spaghetti squash *(*sqh*) encodes the myosin regulatory light chain in *Drosophila.* Notch appeared to be unaltered in the wing imaginal discs obtained from null mutant of *spaghetti squash* (Fig. S4C) indicating that the absence of myosin heavy chain (encoded by *zip*) is mainly responsible for the accumulation of Notch receptor at the cell surface (Fig. S4).

### Over-expression of *zip* rescues *Notch* loss-of-function phenotype and this rescue is facilitated by motor domain of Zip

**Fig. 5:**
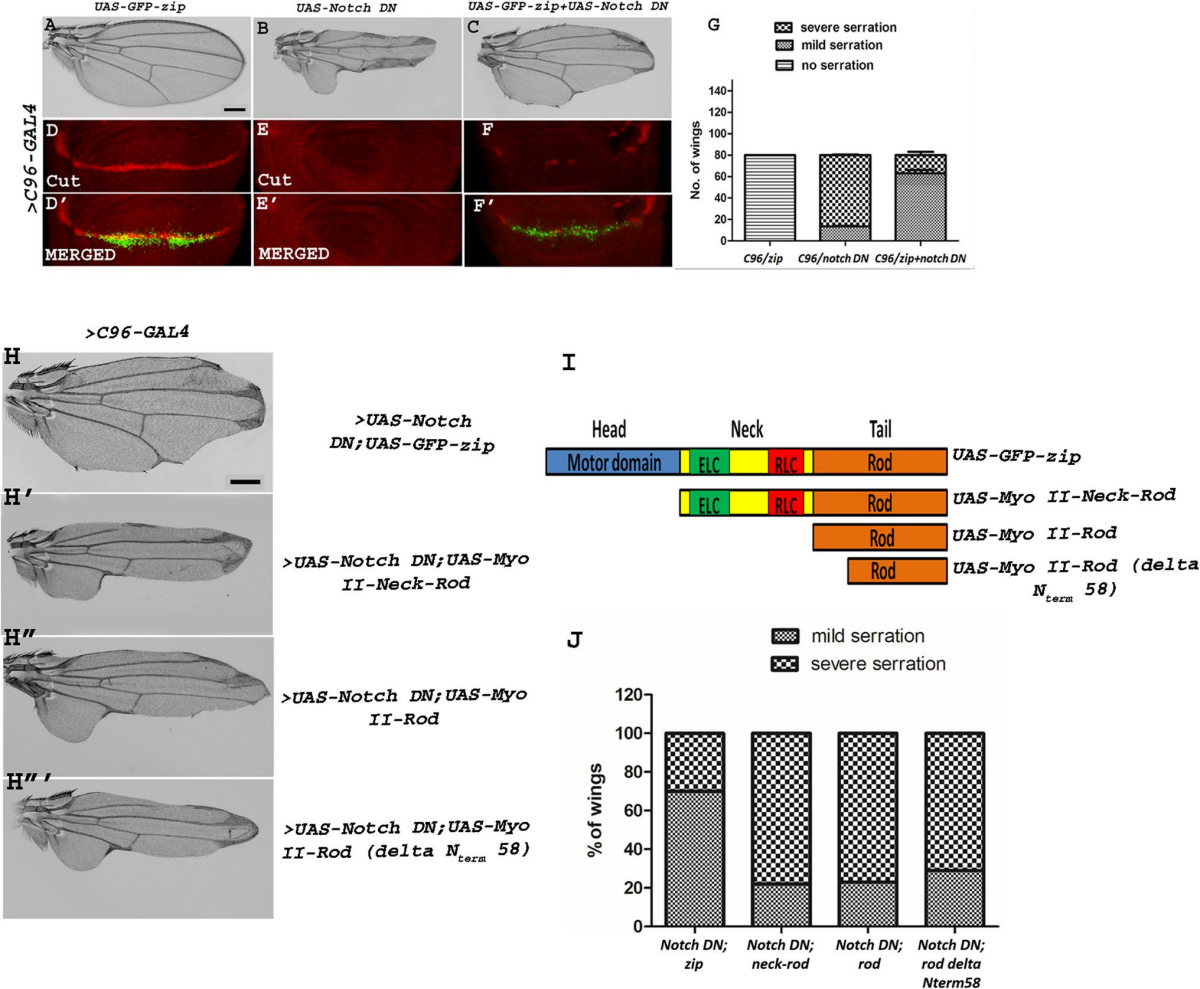
Over-expression of *zip* rescues *Notch* loss-of-function phenotype. **A**–**C** *C96-GAL4*-driven expression of *UAS-Notch-DN* leads to severe wing-nicking phenotype (**B**) that is rescued upon expression of *zip* in the same background (**C**). Over-expression of *zip* alone results in wild-type wing (**A**). Scale bar: 3 cm. **G** Graph representing the number of wings showing rescue in wing serration on co-expressing *zip* with *Notch-DN* using *C96-GAL4*. The genotype of the flies mentioned on the *X*-axis of the graph are as follows: *C96-GAL4/UAS-GFP-zip, C96-GAL4/UAS-Notch-DN,* and *C96-GAL4/UAS-GFP-zip* + *UAS-Notch-DN.* **D**–**F**′ Representative wing discs showing the expression pattern of Cut. Downregulating Notch in *C96-GAL4* region resulted in loss of expression of Cut on the DV boundary (**E**) which was mildly rescued upon over-expressing *zip* in the same background (**F**). Over-expression of *zip* alone resulted in the expression of Cut similar to wild type (**D**). **D**′, **F**′ Merged images showing the expression pattern of Cut under *C96-GAL4*-driven *GFP-zip* and *GFP-zip* with *Notch DN*, respectively. Similarly,** E**′ represents the merged image showing the expression pattern of Cut under *C96-GAL4*-driven *UAS-Notch DN*. Absence of GFP in this image denotes the absence of GFP-zip. Scale bar: 20 μm. **H**–**H**‴ Zip interacts with Notch via motor domain. **H** Co-expression of full-length *UAS-zip* with *UAS-Notch-DN* using *C96-GAL4* lead to rescued wing serration caused by Notch loss of function alone. However, this rescue failed to occur when *UAS-Notch-DN* was co-expressed with *UAS-Myo II Neck Rod* having truncated motor domain (**H**′), *UAS-Myo II-Rod* (**H**″) and *UAS-Myo II-Rod *(*delta N*_*term*_*58*) (**H**‴) having truncated head and neck domain respectively. Scale bar: 3 cm.** I** A depiction of full-length *UAS-GFP-zip* and domain truncation stocks. *UAS-GFP-zip* has all the domains including head domain, neck domain harboring binding sites for essential and regulatory light chains, and rod domain. *UAS-Myo II Neck Rod* has truncated head domain. *UAS-Myo II Rod* has truncated head and neck domains. *UAS-Myo II-Rod *(*delta N*_*term*_*58*) has truncated head, neck and deletion of 58 amino acids of Rod domain*.* **J** Graphical representation of the percentage of wings showing mild and severe wing serration upon co-expression of *UAS-Notch-DN* with full-length *zip* and its truncation domains. The genotype of the flies mentioned on the *X*-axis of the graph is as follows: *C96-GAL4/UAS-Notch-DN* + *UAS-GFP-zip*, *C96-GAL4/UAS-Notch-DN* + *UAS-Myo II Neck Rod*, *C96-GAL4/UAS-Notch-DN* + *UAS-Myo II Rod*, *C96-GAL4/UAS-Notch-DN* + *UAS-Myo II-Rod *(*delta N*_*term*_*58*)

Through epistatic interaction studies, here we wanted to check whether over-expressing Zip in the compromised Notch background can rescue *Notch* loss-of-function phenotypes. At this end, we over-expressed Zip in larval wing imaginal discs of *UAS-GFP-zip* individuals and reduced the expression of Notch in the same tissue by dominant-negative Notch using *C96-GAL4* driver. Notch, upon being downregulated under *C96-GAL4*, yielded a highly serrated wing phenotype which was significantly rescued by over-expressing *zip* in the same background (Fig. 5A–C). This rescue in the wing serration upon upregulating Zip in Notch DN background using *C96 GAL4* has been shown via a bar graph (Fig. 5G) where massive nicking of the wing margin has been referred to as “severe serration” (e.g., Fig. 5B) and a lesser nicking of the wing margin has been referred to as “mild serration” (e.g., Fig. 5C). Here, the experiment was performed in two batches (Batch1 and Batch2) where a total number of 80 wings were examined for each case. In batch 1, *C96 GAL4*-driven *UAS-GFP-zip* yielded all the wings with no serration, *UAS-Notch DN* yielded 66 wings with severe serration and 14 wings with mild serration, *UAS-GFP-zip* + *UAS-Notch DN* yielded 66 mildly serrated wings (rescued) and 14 severely serrated wings. In batch 2, *C96 GAL4*-driven *UAS-GFP-zip* yielded wild type wings with no serration, *UAS-Notch DN* displayed 67 wings with severe serration and 13 wings with mild serration, *UAS-GFP-zip* + *UAS-Notch DN* yielded 60 mildly serrated wings (rescued) and 20 severely serrated wings.

This was further validated in wing discs where we wanted to assess the expression of the Notch signaling target Cut upon over-expressing *zip* in the lowered Notch background. Over-expression of *zip* alone using *C96-GAL4* resulted into the expression of Cut almost like wild type (Fig. 5D). Downregulating Notch signaling using dominant-negative form of Notch under *C96-GAL4* resulted in complete loss of Cut staining in the wing discs (Fig. 5E). However, the loss of Cut expression was mildly rescued (Fig. 5F) upon over-expressing *zip* in the same background indicating an important role of Zip in Notch signaling (Fig. 5D–F).

Subsequently, we wanted to investigate the functional domain of Zip that is responsible for the rescue of the serrated wing phenotype caused by Notch loss-of-function. Several studies suggest that not all the domains of non-muscle myosin II are required for various biological processes [40]. It has been observed that some processes require the contractility based function of non-muscle myosin II while other processes take place normally even if the motor domain is perturbed by amino acid replacements [41–43]. Hence, we wanted to characterize the functional domain involved in Notch and Zip interaction that would subsequently lead to a significant rescue of the nicked wing phenotype of Notch dominant-negative individuals. In *Drosophila,* separate genes encode each subunit of non-muscle myosin II: *zip* encodes the heavy chain (zip/MyoII), *spaghetti squash* encodes the regulatory light chain (sqh/RLC) and *mlc-c* encodes the essential light chain (mlc-c/ELC) [16, 17, 40, 44]. Zip i.e., each individual heavy chain consists of (1) a globular N-terminal motor or head domain (~ 800 amino acids) that contains the ATP and actin-binding sites; (2) a neck domain (~ 50 amino acids) composed of two IQ motifs that bind one ELC and one RLC; (3) a coiled coil or rod domain (~ 1100 amino acids) composed of heptad repeats; and (4) a short C-terminal segment, termed the tailpiece of ~ 34–47 amino acids in length [45]. Truncation alleles with truncated domains of non-muscle myosin II was used for the study of the interaction between Zip domain and Notch. *UAS-Myo II-Neck-Rod* truncation allele lacks only the motor domain, *UAS-Myo II-Rod* encompasses the entire rod domain, thus lacking the motor domain and the neck region and *UAS-Myo II-Rod *(*delta N*_*term*_*58*) lacks only the first 58 amino acids of the rod domain along with motor and neck domains (Fig. 5I) [40]. It was observed that no rescue of the wing-nicking phenotype could occur when *UAS-Notch-DN* was co-expressed with the truncated allele, *UAS-Myo II-Neck-Rod*, *UAS-Myo II-Rod* and *UAS-Myo II-Rod *(*delta N*_*term*_*58*) (Fig. 5H′–H‴). Our findings indicated that the truncation of motor domain fails to rescue the nicked wing phenotype caused by Notch loss-of-function suggesting that motor domain is indispensable for the function of Zip in regulation of Notch.

### *z*ip synergises with Notch

It is apparent from genetic interaction experiments that *zip* modulates Notch signaling activity. Hence, we wanted to explore the integrative effect of Zip in Notch signaling. To investigate this, we over-expressed *Notch-FL* and *zip* together using *vg-GAL4* driver. Over-expression of *zip* alone resulted in wild-type wing phenotype and over-expressed *Notch-FL* alone resulted in multiple wing phenotypes, such as wing crumpling, wing blisters, ectopic outgrowth, vein disorganization, and ectopic marginal bristles. Over-expression of both *zip* and *Notch-FL* together resulted in enhancement of only Notch-FL over-expression induced wing phenotypes (Fig. S5A–C). Massive wing disorganization, such as wing duplication, large wing blisters, severe vein disorganization, etc., was observed upon co-expression of *Notch-FL* and *zip* (Fig. S5C).

**Fig. 6:**
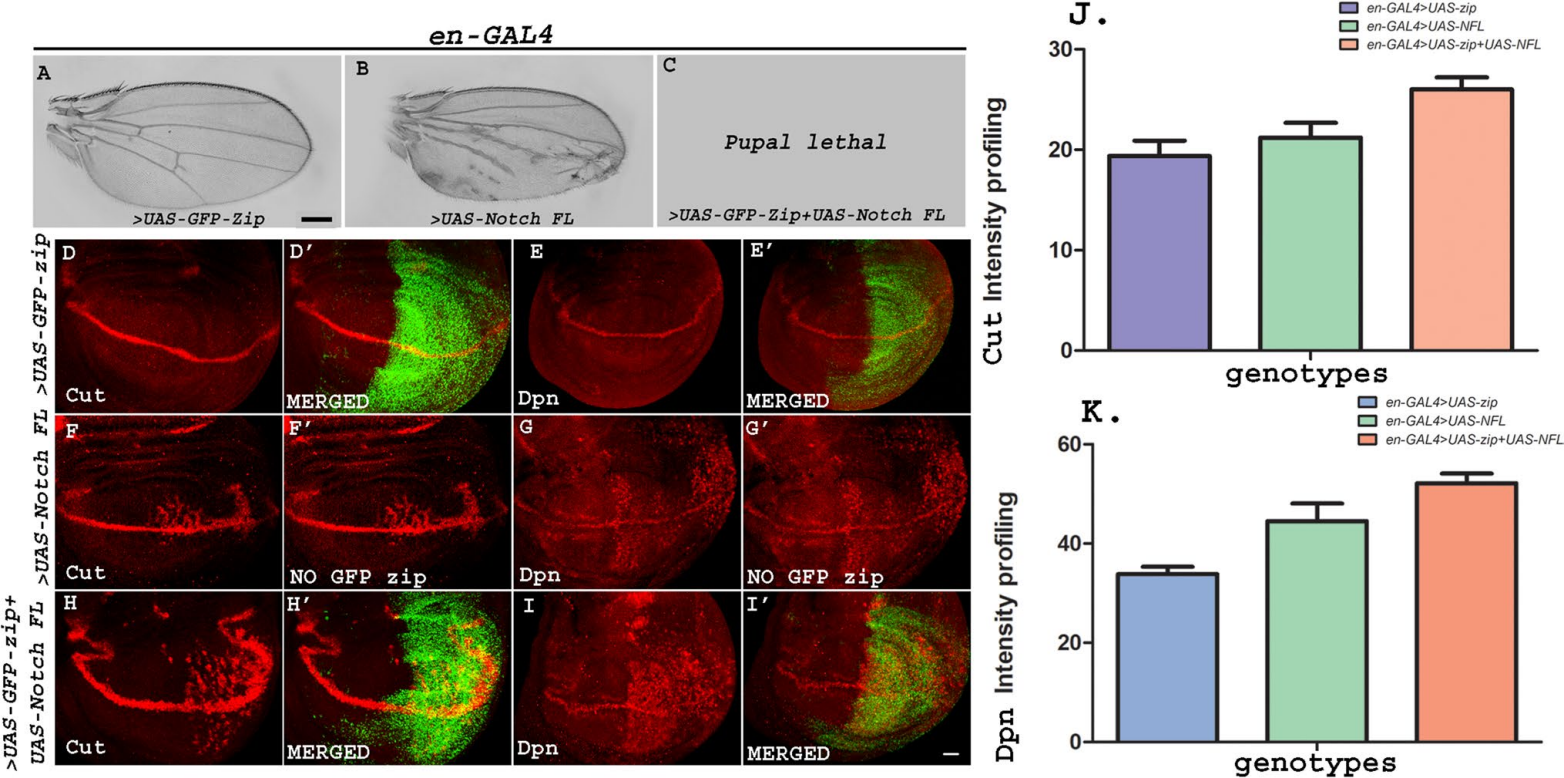
**A**–**I**′ Zip synergises with Notch-FL to lead to pupal lethality and increase in the ectopic expression of Cut and Wg on D/V boundary. **A**–**C** Zip synergy with Notch lead to pupal lethality. **A** Over-expression of *zip* by *en-GAL4* resulted in wild type wings whereas *Notch-FL* over-expression in the posterior domain resulted in disruption of wing morphology with shortening and thickening of fourth and fifth vein, presence of extra vein material, wing crumpling, and loss of cross veins (**B**). Flies failed to emerge from the pupal case when *UAS-Notch–FL* was co-expressed with *UAS-zip* resulting in 100% pupal lethality (**C**). Scale bar: 3 cm. **D**–**I**′ Representative wing discs show the expression patterns of Cut and Dpn. Ectopic expression of Cut (**H**) and Dpn (**I**) gets enhanced upon co-expression of *zip* with *Notch *(*FL*) under *en-GAL4* in contrast to over-expressed *zip* that shows wild-type expression of Cut and Dpn (**D**, **E**) and over-expressed *Notch-FL* that shows mild ectopic expression of Cut and Dpn (**F**, **G**). **D**′, **H**′ are the merged images showing expression pattern of Cut under *en-GAL4*-driven expression of *GFP-Zip* and *GFP-zip* with *Notch-FL*, respectively. Similarly, **E**′, **I**′ are the merged images showing the expression pattern of Dpn using *GFP-zip* and *GFP-zip* with *Notch-FL,* respectively, in the posterior region. **F**′, **G**′ panel represent the expression pattern of Cut and Dpn respectively under *Notch *(*FL*) driven by *en-GAL4.* Absence of GFP in this image represents absence of GFP-zip. **J**, **K** Graphs representing the intensity profiling of Cut and Dpn in *en-GAL4* > *UAS-zip, en-GAL4* > *UAS-Notch FL and en-GAL4* > *UAS-zip + UAS-Notch FL*. Scale bar: 20 μm

In order to check the synergistic effect of Notch-FL and Zip on the wing phenotype using other GAL4 driver line, we co-expressed *UAS-Notch-FL* and *UAS-GFP-zip* in the posterior wing compartment using *en-GAL4* driver. Zip yielded almost wild type wings when over-expressed using *en-GAL4*. Notch-FL over-expression in the posterior domain resulted in multiple wing phenotypes including fifth vein shortening, extra vein material, wing crumpling and blisters. When Zip was co-expressed with Notch-FL, it culminated in 100% pupal lethality, thus confirming a significant synergistic effect of Notch and Zip (Fig. 6A–C).

To further validate the synergistic interaction between Notch and Zip, we analyzed the levels of Notch targets, Cut and Dpn. Over-expressing GFP-tagged *zip* alone using *en-GAL4* driver did not result in any significant increase in the expression of Cut and Dpn. Over-expression of only *Notch-FL* in the posterior compartment of wing disc resulted in increased Cut and Dpn expression. However, co-expression of *zip* and *Notch-FL* under *en-GAL4* resulted in an elevated level of Cut and Dpn in the posterior domain of wing imaginal discs (Fig. 6D–I′). Image J was used for the intensity profiling. A total of 23 discs were examined and all of them showed consistent results. 6 discs were used for the quantification. Mean intensity for Cut expression in *en-GAL4-driven* > *UAS-GFP-zip* was 19.4, for *UAS-Notch FL* was 21.2 and for *UAS-GFP-zip* + *UAS-Notch FL* was 26. Mean intensity for Dpn expression in *en-GAL4-driven* > *UAS-GFP-zip* was 34, for *UAS-Notch FL* was 44.5 and for *UAS-GFP-zip* + *UAS-Notch FL* was 52.17.

At this point, it was interesting to explore the synergistic interaction between Zip and activated Notch. Hence, we explored the co-operative effect of *zip* with processed *Notch *(*Notch-ICD*). To analyze this, we co-expressed both *Notch-ICD* and *zip* using *C96-GAL4* driver. *C96-GAL4*-driven *zip* alone did not show any phenotype, whereas *C96-GAL4*-driven *Notch-ICD* resulted in wing margin defects with irregular marginal bristles resulting in mild crumpling of the wing. When *Notch-ICD* and *zip* were co-expressed together with *C96-GAL4* driver, it resulted in increased irregular marginal bristles and severely crumpled wings (Fig. S5D–F).

In order to determine that GAL4 is not a limiting factor in our experimental set-up and there is a similar level of expression of transgenes downstream to single copy of UAS (*UAS-GFP zip/UAS-Notch FL/UAS-Notch ICD*) in control case and double copy of UAS (*UAS-GFP zip* + *UAS-Notch FL*) in experimental condition, we performed immunoblotting to check the expression level of Zip and Notch in protein lysates from *UAS-GFP-Zip, UAS-Notch FL, UAS-Notch FL* + *UAS-GFP Zip* and *UAS-Notch ICD* + *UAS-GFP-Zip* driven with *GMR-GAL4*. A similar level of Zip and Notch expression was observed in the transgenes with single UAS and double UAS copy (Fig. S1D′–F′). The graphs depicting a similar band intensity suggest that irrespective of single or double copy of UAS, the expression level of transgenes is almost same in all the cases and that GAL4 is not a limiting factor here (Fig. S1D″–F″). Similarly, we also analyzed the GFP-Zip intensity between *en-GAL* > *UAS-GFP-zip* and *en-GAL* > *UAS-GFP-zip* + *UAS-NotchFL* condition (Fig. 6D′, H′; E′, I′) to rule out the potential GAL4 dilution effect. The mean intensities of GFP-Zip between respective genotypes were observed to be 32.5 in Zip alone condition and 37.5 in Zip co-expression with Notch FL. The difference in the intensities between both the genotypes was calculated to be non-significant (p > 0.05), thus indicating that there has been no case of GAL4 dilution effect in our studies (Fig. S1F″).

### Interactivity of Zip and activated Notch results into hyperplasia, high mitotic activity index (MAI) and compromised epithelial integrity

**Fig. 7:**
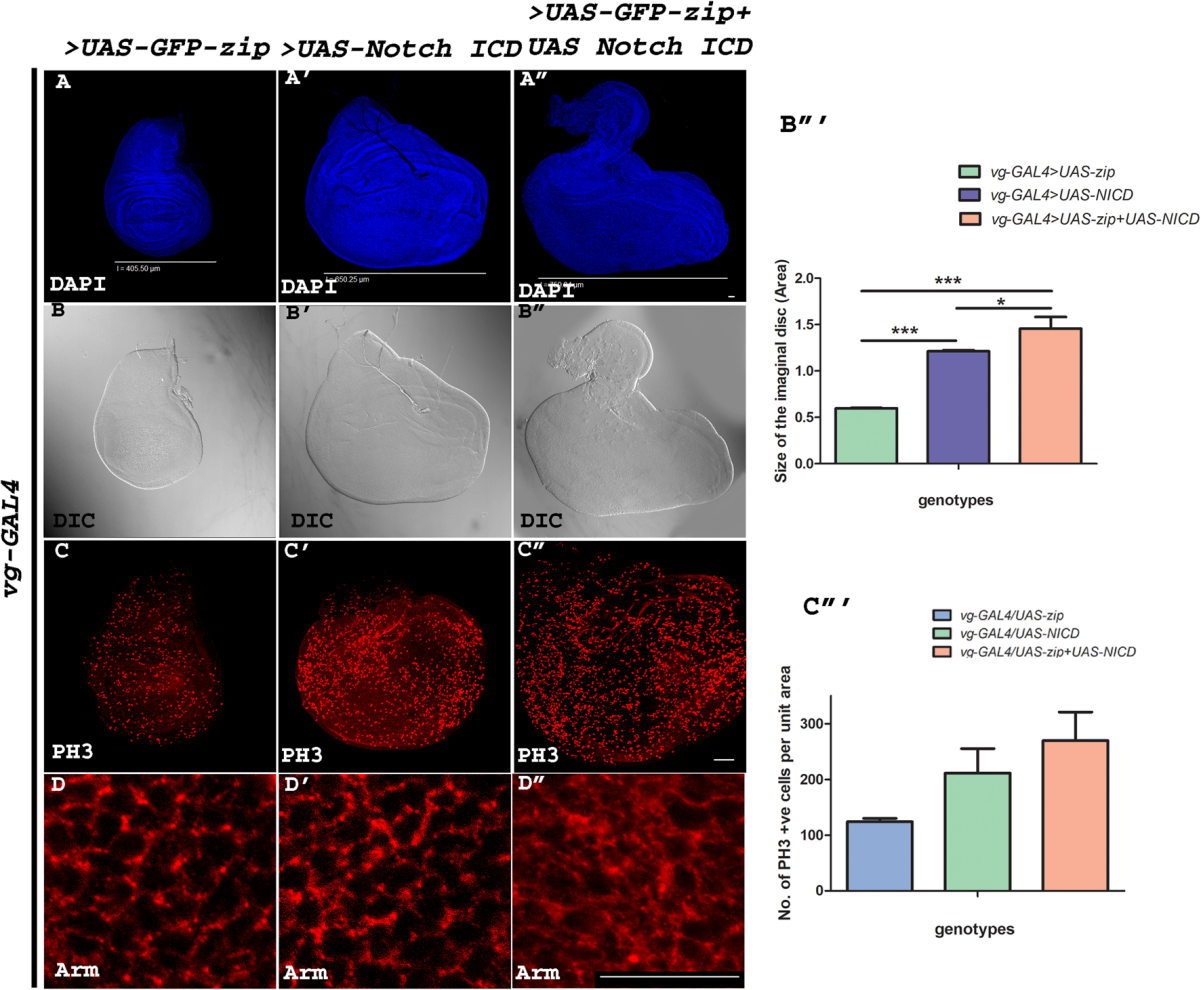
**A**–**D**″ Co-expression of Zip and Notch ICD results into hyperplasia, high mitotic activity index (MAI) and compromised epithelial integrity. **A**–**B**″ Representative wing discs showing the wild type wing morphology when *UAS-GFP-zip* was over-expressed with *vg-GAL4* (**A**, **B**). Wing morphology was observed to be hyperplastic when *UAS-Notch-ICD* was over-expressed with *vg-GAL4* (**A**′, **B**′). Co-expression of *UAS-Notch-ICD* along with *UAS-GFP-zip* using *vg-GAL4* resulted in highly deformed severely hyperplastic tumorigenic wing discs with increase in size (**A**″, **B**″). Scale bar in the panels **A**, **A**′ and **A**″ represents the size of the discs with respective genotypes. **C**–**C**″ Representative wing discs showing mitotic activity index similar to wild type upon over-expressing Zip in vestigial domain (**C**). An increased mitotic activity index was observed upon over-expressing Notch ICD by *vg-GAL4* (**C**). However, PH3 puncta representing MAI was drastically increased upon co-expressing Notch ICD with Zip in vestigial compartment (**C**″). **D**–**D**″ Armadillo staining showing the status of adherent junctions when *zip* alone, *Notch-ICD* alone and *Notch ICD* along with *zip* was co-expressed using *vg-GAL4*. Epithelial integrity was observed to be almost wild type when *zip* and *Notch ICD* were over-expressed alone. However, highly disrupted tissue integrity was found upon co-expression of *UAS-Notch-ICD* and *UAS-zip* using *vg-GAL4* (**D**″). **B**‴ Graphical illustration of the size of the imaginal disc in *vg-GAL4* > *UAS-zip, vg-GAL4* > *UAS-Notch ICD* and *vg-GAL4* > *UAS-zip* + *UAS-Notch ICD* imaginal discs. One-way analysis of variance (ANOVA) followed by Tukey’s multiple comparison test was conducted to calculate the *p* values (*p < 0.05, ***p < 0.001). **C**‴ Graph representing the approximate number of PH3 positive cells per unit area in *vg-GAL4* > *UAS-zip, vg-GAL4* > *UAS-Notch ICD* and *vg-GAL4* > *UAS-zip* + *UAS-Notch ICD* imaginal discs. Scale bar: 20 μm

The integration of other genes with Notch signaling is known to modulate its downstream activity [46–48]. To further explore and dissect the downstream changes mediated by Zip upon integration with Notch signaling, we co-expressed *zip* with *Notch-ICD* in the ventral domain of the wing disc using *vg-GAL4*. We have already shown the role of Zip in modulating Notch signaling outcome by interacting with the Notch-FL. Here we wanted to explore the effect of Zip over-expression in activated Notch-mediated hyperproliferation. Over-expression of *zip* alone does not cause any significant changes in wing disc morphology and over-expression of *Notch-ICD* results in hyperplastic wing disc with deformed morphology. When *zip* and *Notch-ICD* both were co-expressed, the severity of tumorous phenotype was much more increased in terms of size and morphology (Fig. 7A–B″). Here, 3 discs of each genotype were analyzed to determine the size of the tumorous wing discs. To determine the proliferation activity in these discs, mitotically active cells were checked using Phosphohistone H3 (PH3) staining. An increase in the number of mitotically active cells was apparent when *zip* was co-expressed with *Notch-ICD* (Fig. 7C–C″)*.* At this end, 3 discs from each genotype were analyzed to determine the number of PH3 positive cells.

Further, we examined the cell–cell junction integrity to measure the severity of tumorous-phenotype. We investigated the status of Armadillo (Arm), β-catenin homologue in fly, which is an important component of the cytoskeleton and is required to maintain the integrity of the tissue. Cell–cell junction integrity is maintained when Zip or Notch-ICD are expressed individually. However, it was observed that *zip* upon co-expression with *Notch-ICD* in the vestigial domain significantly deregulated the expression of junctional molecule as evident by the disrupted and diffused expression pattern of Arm (Fig. 7D–D″). The compromised integrity of the junctional molecule in the hyperplastic discs indicates the combinatorial effect of Zip and Notch in Notch-mediated tumorigenesis.

To check whether Notch has any role in the regulation of Zip activity, we examined the phosphorylation status of myosin regulatory light chain in the wing imaginal discs of Notch over-expressed (Notch-induced hyperplastic) condition in ventral domain using *vg-GAL4* (*vg-GAL4* > *UAS-Notch ICD*) and down-regulated condition at D/V boundary using *C96-GAL4* (*C96-GAL4* > *UAS-Notch DN*). Anti-phospho-myosin regulatory light chain 2 antibody was used for studying the phosphorylation status of the light chain. Oregon R served as the control. No alteration in the phosphorylation status of myosin regulatory light chain could be observed in Notch perturbed condition compared to the control. This indicated that Notch does not modulate Zip activity (Fig. S6A–C″).

We also examined the status of Notch ligand Delta in Notch hyperplastic condition and observed that Delta was unaltered. However, perturbation in the expression pattern of Delta occurred mainly due to the disrupted tumor morphology of the wing disc from Notch over-expressed condition compared to the control (Fig. S6D–E″).

### Zip induces JNK-mediated invasiveness in tumors caused by activated Notch

To determine whether the Notch-induced tumorous phenotypes become more aggravated in presence of over-expressed Zip, we analyzed the expression of Matrix metalloproteinase 1 (MMP1) that disintegrates the ECM in Notch and Zip co-expressing wing discs.

**Fig. 8:**
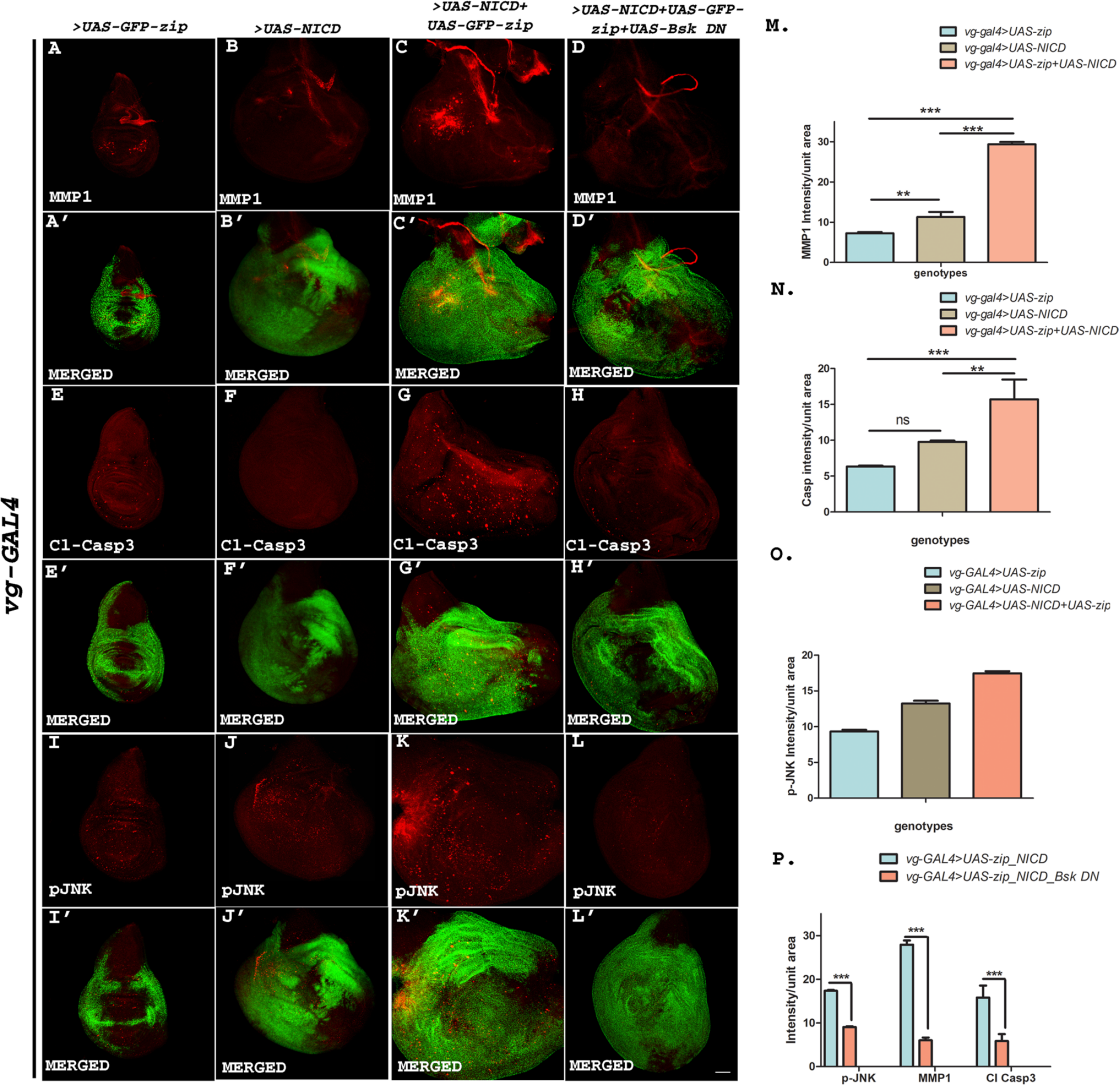
**A**–**P** Zip induces JNK mediated invasiveness in tumors caused by Notch. **A**–**C** Representative wing discs showing the status of MMP1 in *zip* over-expressed condition with *vg-GAL4* where the expression level of MMP1 was observed to be almost wild type (**A**). *Notch ICD* when over-expressed in ventral domain results in no MMP1 expression (**B**). However, mmp1 expression was observed to be upregulated when *UAS-Notch ICD* was co-expressed with *UAS-GFP-zip* using *vg-GAL4* (**C**). **A**′–**C**′ Merged images showing the expression status of MMP1 under *vg-GAL4*-driven *UAS-GFP-zip, UAS-Notch-ICD* and *UAS-GFP-zip* along with *UAS-Notch-ICD* respectively.** E**–**G** Wing discs showing the expression level of Cleaved Caspase 3 upon co-expression of Notch ICD along with Zip and Notch ICD and Zip individually. A mild and no expression of Cl Casp3 was observed upon over-expression of *Notch ICD* and *zip* using *vg-GAL4* respectively (**E**-**F**). However, this expression was increased when the two proteins were co-expressed in the proliferated vestigial domain (**G**). **E**′–**G**′ Merged images showing the expression pattern of Cleaved Caspase 3 using *vg-GAL4*-driven *UAS-GFP-zip, UAS-Notch-ICD* and *UAS-GFP-zip* + *UAS-Notch-ICD*. **I**–**K** Representative wing discs showing the status of activated JNK with phospho-JNK staining. **I**, **J** Mild expression of pJNK puncta was observed upon over-expression of *UAS-GFP-zip* and *UAS-Notch-ICD* with *vg-GAL4*. However, an increase in the expression level and puncta size of pJNK was observed upon co-expression of *Notch-ICD* with *zip*. **I**′–**K**′ Merged images showing the expression status of pJNK with *vg-GAL4*-driven *UAS-GFP-zip, UAS-Notch-ICD* alone and *UAS-GFP-zip* together with *UAS-Notch-ICD.* **D**, **H**, **L** Wing discs showing the suppressed expression status of MMP1 (D), Cleaved Caspase 3 (H) and pJNK (L) in vestigial domain upon blocking JNK using *UAS-Bsk-DN* in the background with co-expressed *UAS-Notch-ICD* and *UAS-GFP-zip*. **D**′, **H**′, **L**′ represents the merged images showing the expression status of MMP1, Cleaved Caspase 3 and pJNK with co-expressed *UAS-Notch-ICD, UAS-GFP-zip* and *UAS-Bsk-DN* using *vg-GAL4*. (M-P) Graphical representation of MMP1, Cleaved Caspase 3, and pJNK intensity in *vg-GAL4*-driven *UAS-GFP-zip, UAS-Notch-ICD, UAS-GFP-zip* + *UAS-Notch-ICD* and *UAS-GFP-zip* + *UAS-Notch-ICD* + *UAS-Bsk-DN.* (M and N) One-way analysis of variance (ANOVA) followed by Tukey’s multiple comparison test was performed to calculate *p* values (ns > 0.05, **p < 0.01, ***p < 0.001). Unpaired t test was performed to calculate *p* values (***p < 0.001). Scale bar: 20 μm

A prerequisite for invasiveness or metastasis to occur is the penetration of the surrounding extracellular matrix (ECM) stroma and epithelial basement membranes by tumor cells [49]. The basement membrane and ECM components are degraded by enzymes, matrix metalloproteinases (MMPs) [50, 51]. Various human tumors display increased MMP expression in correlation with metastasis [52–54]. Hence, we studied the expression levels of MMP1 in the tumorous discs induced by over-expression of Notch alone or Notch and Zip together. No significant expression of MMP1 was observed in the case of wing discs over-expressing only activated Notch (also seen earlier by [47, 55]. Similarly, Zip over-expressed non-tumorous disc also showed no expression of MMP1. However, a high level of MMP1 was detected when *zip* was co-expressed with *Notch-ICD* (Fig. 8A–C).

As such caspase activation is a hallmark of cell death, however recent studies also suggest the role of these proteases in non-apoptotic functions [56, 57]. It has been reported that caspases have a functional role during tumor invasion and metastasis other than their cell death-related function [58]. These non-apoptotic roles have indicated that caspases can get activated without initiating apoptotic cascade leading to the cleavage of specific cellular substrates. One of the non-apoptotic roles of caspases being recently investigated includes controlling cell cycle and driving proliferation leading to cell survival [59, 60]. It has been demonstrated that apical caspase, cleaved caspase-3 can lead to proliferation of the cells via the activation of the JNK pathway which subsequently drives the expression of MMP1 [48, 61]. To examine the role of caspases in Notch and Zip co-expressed tumor, we examined the levels of cleaved caspase-3 in the vestigial domain of the wing discs over-expressing Notch and Zip. A high level of caspase expression was observed in the proliferated vestigial domain of the Notch-Zip co-expressed discs in comparison to the wing disc expressing Notch or Zip alone. This showed that the activation of caspases is linked to the proliferation resulting due to the collaboration of Notch and Zip (Fig. 8E–G). An elevated expression level of caspases leads to the activation of JNK pathway that consequently lead to MMP activation and proliferation [54, 57, 58, 61, 62]. Therefore, we also wanted to explore the phosphorylation status of active JNK (Bsk) in the tumors where Notch and Zip were co-expressed. At this end, we performed immunostaining of the wing imaginal discs with a phospho-JNK-specific antibody recognizing the active form of JNK. In the case of over-expression of *Notch* and *zip* alone, there was no activation of JNK. However, JNK was found to be hyper-phosphorylated in the vestigial domain when *zip* was co-expressed with *Notch-ICD,* indicating the involvement of JNK in inducing invasive tumor (Fig. 8I–K).

To further validate that JNK is the key player in causing aggressive tumor due to Notch and Zip synergy, we blocked the JNK signaling in the same background using a dominant-negative form of Basket (*UAS-Bsk DN*)*.* By abrogating JNK signaling in the background co-expressing *Notch-ICD* and *zip*, we wanted to see if we can rescue the hyperproliferative activity by comparing the size, and metastatic behavior by MMP1 expression levels in these tumors. Immunostaining experiments were performed to analyze the level of phosphorylated JNK, MMP1, and Cleaved caspase3 in this background. As expected, the JNK signaling was significantly reduced upon expression of *Bsk-DN* (Fig. 8L). Further, the level of MMP1 and Cleaved caspase3 was also significantly reduced to near wild type levels in Notch-Zip co-expressed discs when JNK was blocked (Fig. 8D, H). These results confirmed the role of JNK in inducing hyper-proliferation and invasiveness in the tumorigenic discs resulting due to Notch and Zip synergy.

Graphs were plotted to show the intensity of the expression of MMP1, Cleaved Caspase3 and p-JNK. A total of 3 discs were used for the quantification purpose for every genotype. Mean intensity for MMP1 for different genotypes was observed as follows: vg-GAL4-driven *GFP-zip*: 7.25, *Notch ICD*: 11.29, *GFP-zip* + *Notch ICD*: 29.356 and *GFP-zip* + *Notch ICD* + *Bsk DN:* 6.023. Mean intensity for Cl Casp3 for different genotypes was observed as follows: vg-GAL4-driven *GFP-zip*: 6.334, *Notch ICD*: 9.77, *GFP-zip* + *Notch ICD*: 15.711 and *GFP-zip* + *Notch ICD* + *Bsk DN:* 5.83. Mean intensity for p-JNK for different genotypes was observed as follows: vg-GAL4-driven *GFP-zip*: 9.32, *Notch ICD*: 13.25, *GFP-zip* + *Notch ICD*: 17.47 and *GFP-zip* + *Notch ICD* + *Bsk DN:* 9.07.

## Discussion

Here we present evidence that non-muscle myosin II (NM II) Zip physically and genetically interacts with Notch. Down-regulation of Notch signaling targets either using RNAi or through dominant-negative over-expression line clearly showed that Zip positively regulates Notch signaling. Synergistic interactions between Notch and Zip also indicated that Notch is positively regulated by Zip. Upregulation of downstream targets of the Notch pathway, Cut, and Dpn, was detected when Zip and Notch were co-expressed. Here we showed that Zip integrates with processed Notch to cause invasive tumor of the wing discs. Increased activity of JNK signaling downstream to Notch/Zip collaboration was evident by hyperphosphorylated Basket (orthologue of JNK) in the hyperplastic discs. Thus, we postulate that Notch/Zip synergy converges on JNK signaling that ultimately regulates invasive behavior of the tumorous discs.

Earlier it was proposed that DSL ligand binding to heterodimeric Notch receptor in the plasma membrane induces proteolytic cleavage (S2) via a disintegrin and metalloprotease (ADAM), which facilitates γ-secretase proteolysis (S3) within the transmembrane region to release the Notch intracellular domain (NICD) [6, 63]. Later on, it was shown that in canonical ligand-dependent Notch pathway, ligand binding and subsequent endocytosis of the NECD-bound ligand into the ligand-expressing signal-sending cell generates pulling-force on the extracellular fragment of Notch, which causes some conformational changes in the Notch receptor and consequently allows S2 cleavage to occur. Thus, NECD dissociation is not a consequence of S2 site cleavage, but rather that dissociation occurs first, and subsequently allows for S2 site cleavage by ADAMs [7, 64, 65]. Interestingly NM II plays critical role in clathrin-mediated endocytosis which is important for receptor-mediated signaling. It has been shown that clathrin-mediated endocytosis is actomyosin-dependent [38, 66]. Endocytosis is required for ligand-dependent Notch activation in both signal-sending and receiving cells. Recently, it was shown that NM II activity is required for robust Notch signaling and NM II is important for signaling in both signal-sending and receiving cells [23]. NM II contractility plays important role in Notch signaling and NM II-mediated mechanotransduction via which forces generated within the actomyosin cytoskeleton influence signaling. Actomyosin contractility in cells having extensive lateral contacts with other cells has been shown to promote Notch activation and it was postulated that endocytosis and myosin-dependent pulling may both contribute to force-dependent Notch activation in these cells [23]. Here we showed that membrane accumulation of Notch and early endosomal marker Rab5 occurred in Zip loss-of-function condition which clearly indicates that NM II Zip plays important role in endocytosis of Notch. Further, large-scale proteomic analysis based on co-immunoprecipitation of GFP-tagged Zip interacting proteins using anti-GFP antibody identified several proteins of endocytic machinery as interacting partners of Zip along with Notch. Some of these proteins were Clathrin heavy chain, Dynamin, Dynein heavy chain, Kinesin heavy chain, AP-2 complex subunit alpha, Ras-related protein Rab 3, etc. Identification of these components as interacting partners of Zip suggested that Zip may have a major role to play in the endocytosis of Notch receptor in the signal receiving cell. Biological processes, such as cytoskeletal organization, establishment of vesicle localisation, receptor-mediated endocytosis, myosin filament assembly, and receptor internalization, was observed to be highly enriched in the proteomic network thus reinforcing role of Zip in Notch receptor endocytosis.

Further, presence of non-muscle myosin IIs at the nuclear periphery and its colocalization with the linker of nucleoskeleton and cytoskeleton (LINC) protein Nesprin2 and apical actin caps suggests that it plays role in transmission of cytoplasmic signals to the nucleus [67, 68]. Recently it has been suggested that nuclear envelope proteins are involved in regulation of gene expression by signal transmission from the cytoskeleton to the nucleus [69]. Here we have shown the synergistic interaction of Zip with FL-Notch as Notch downstream targets were ectopically expressed when these two proteins were co-expressed in larval discs. We have also presented the combinatorial effect of Zip and activated Notch in Notch-mediated tumorigenesis. Recently it has been shown that NMIIs colocalize at the perinuclear area and modulate the expression of genes associated with cancer progression and it was postulated that NMIIs may act as a mechanotransducer during tumor progression [67]. We have also identified cyto- and nucleo-skeleton components that aid in nuclear transmission of signals from the cytoplasm to nucleus as interacting partners of Zip in our MS analysis. These components included Nesprin-1 and Nesprin-2, nuclear anchorage protein, Lamin, nuclear pore complex proteins, etc. The underlying mechanism is unknown at this point, but it is possible that similarly, Zip may enhance the Notch signaling activity through its mechanotransduction function.

There is an emerging realization that mutations in Notch gene and aberrant Notch signaling are crucial factors in tumor initiation and progression. It has been shown that depending on the cellular context, Notch behaves as an oncogene or tumor suppressor gene, and a large number of cancers are associated with abnormal Notch functioning. Interestingly, several studies reported that NM II also promotes different types of cancer progression as well as it can also act as a tumor suppressor suggesting its context-dependent function [70]. It has been reported that NMIIs lie downstream of the Wnt signaling pathway and can regulate the local invasion of amoeboid melanoma cells, and its metastatic potential [71–73]. In breast cancer cells, invasiveness was shown to be increased via phosphorylation and switching from NM II C population to NM II B. In cancer stem cells (CSC), NM II B has shown to facilitate squeezing of CSC’s nucleus to pass through tight spaces leading to dissemination [74, 75]. Upregulation of NM IIs in transforming, proliferating cells in the early stages of cancer progression, and knockdown of NM II in these cells leading to altered expression of cancer pathway-related genes further hints at the involvement of NM II in cellular reprogramming [67, 76] Further, several biological processes depend on the proper cellular responses to mechanical forces. Zip–Notch synergy opens up new avenues to study force-dependent Notch activation in different cellular contexts. Further investigation of mechano-transducer Zip-induced Notch signaling activity in different disease conditions including tumorigenesis will advance the development of novel Notch-targeted therapeutics.

### Supplementary Information

Below is the link to the electronic supplementary material.

Supplementary file 1 (DOCX 9565 kb)

## Declarations

### Data availability

The datasets supporting the conclusions of the article are included within the article. Data available upon request.
