# Supplementary material for: Regulation of Notch signaling by non-muscle myosin II Zipper in Drosophila
Source: Cell Mol Life Sci. 2024 Apr 24;81(1):195. doi: 10.1007/s00018-024-05142-1 (PMC11039529; doi:10.1007/s00018-024-05142-1)
Supplement: Supplementary file 1 — (DOCX 9565 kb) [file 18_2024_5142_MOESM1_ESM.docx]

**Supplemental data**

**Figure S1:**


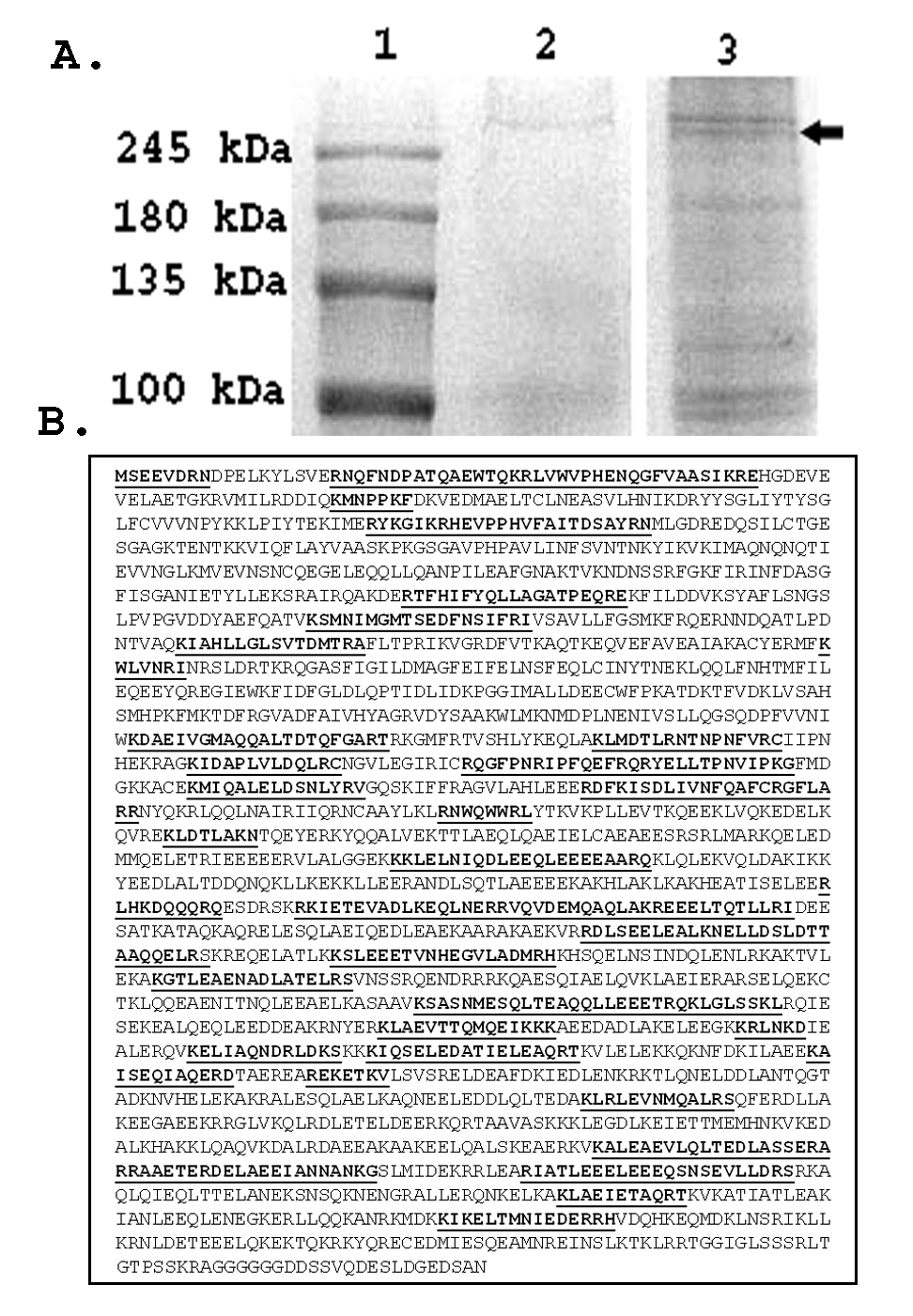


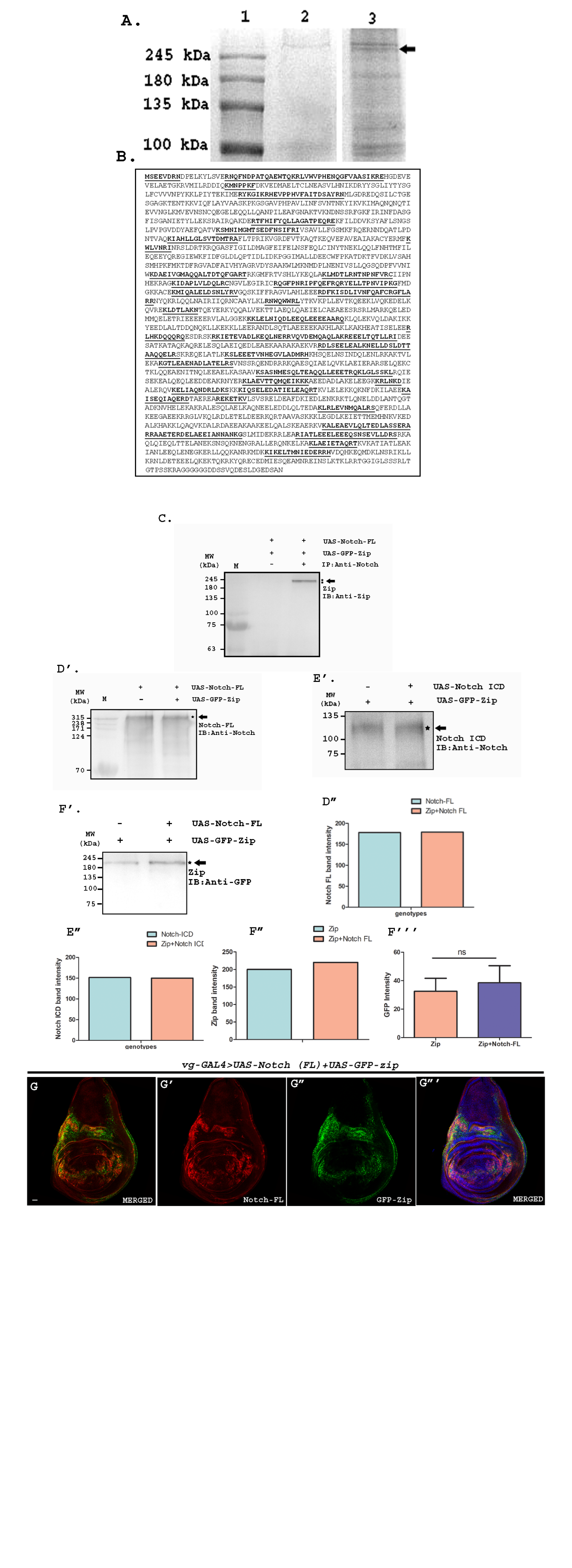


**Figure S1: (A-B)** **Zip was identified as an interacting partner of Notch in mass-spectrometry analysis.** **(A).** SDS-PAGE analysis of proteins after immunoprecipitation with anti-Notch antibody using protein lysate from OregonR (Lane2) and NICD over-expressed (Lane3), followed by staining with Coomassie Brilliant Blue. The band at 245 kDa is exclusively present in lane 3 but not in control lane 2. Lane 1 contains protein marker. **(B)** Amino acid sequence of Zip and of the peptides picked up by MALDI-TOF analysis of excised 245 kDa band present in lane 3 of Figure 1 A (underlined). Thirty four peptides (underlined), some of which are overlapped matched to Zip**. C. Zip was immunoprecipitated with anti-Notch antibody.** Co-immunoprecipitation was carried out with head tissue lysates over-expressing GFP-Zip and Notch FL. M indicates the marker lane, (+) symbol indicates the presence and (-) shows the absence of the specified reagent. Anti-Notch immunoprecipitated endogenous and over-expressed GFP-tagged Zip which was evident as a doublet band upon immunoblotting with anti-Zip antibody. No antibody lane served as the negative control. (**D’-F”)** Immunoblots show the same level of expression of Notch-FL in UAS-Notch-FL and UAS-Notch FL+UAS-GFP-Zip lysate (D’) and a similar level of expression of Notch ICD was observed in both UAS-Notch ICD and UAS-Notch ICD+UAS-GFP-Zip lysate (E’) upon immunoblotting with anti-Notch antibody. Similarly, almost same level of Zip expression was obtained in UAS-GFP-Zip and UAS-GFP-Zip + UAS-Notch FL lysate upon immunoblotting with anti-GFP antibody (F’). **(D”-F”)** Representative graphs showing the band intensity of Notch FL (D”), Notch ICD (E”) and Zip (F”) in respective lysates. **F’’’** Representative graph showing the GFP intensity from *vg-GAL4>UAS-GFP-zip* and *vg-GAL4>UAS-GFP-zip +UAS-Notch FL* (Figure 6). Unpaired t-test was performed to calculate p-values (^ns^p>0.05). **(G-G’’’)** Representative wing imaginal disc showing the expression and colocalisation (G) of Notch-FL and GFP-Zip in the vestigial domain. **Scale Bar: 20µm.**

**Figure S2:**

**
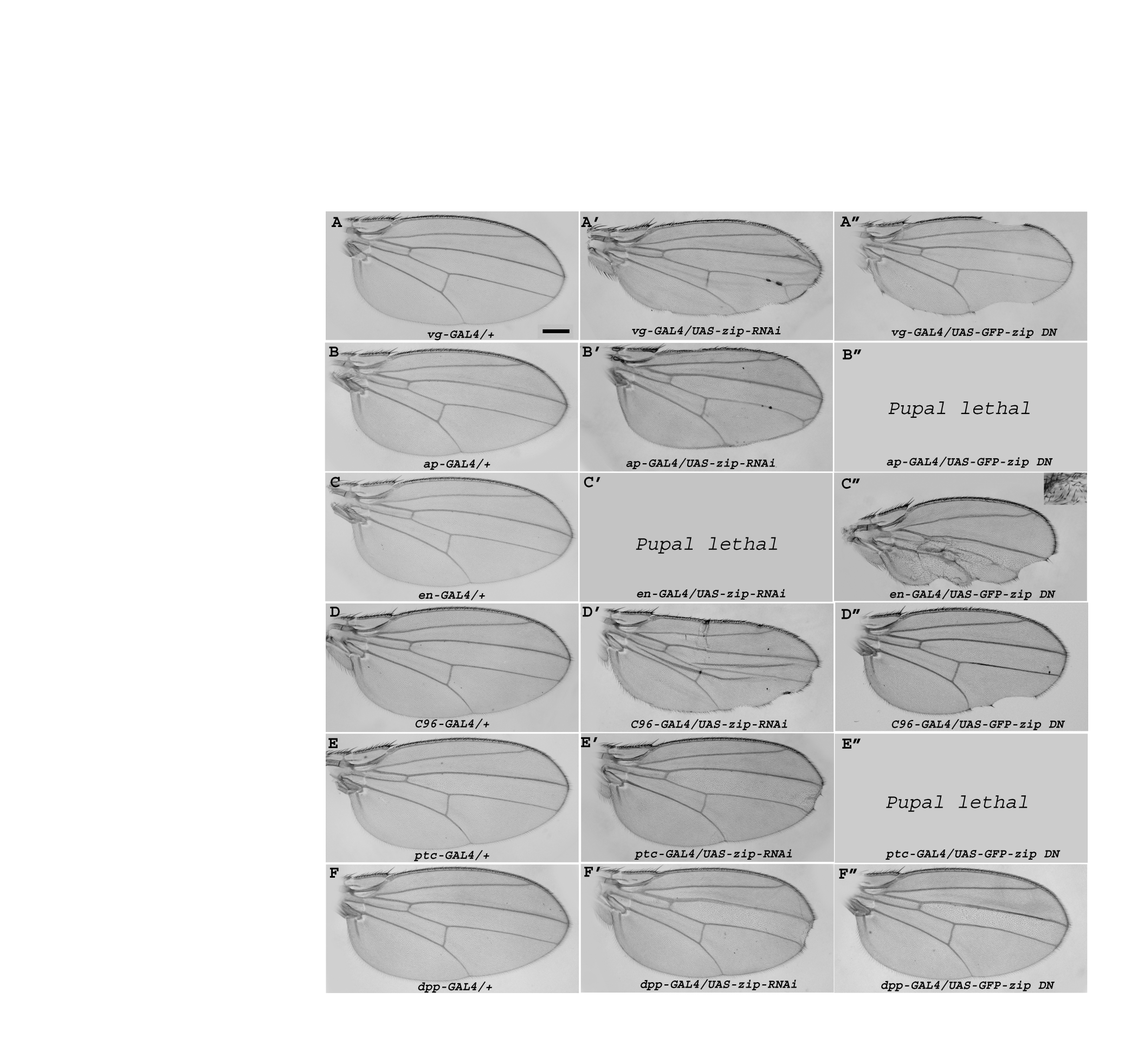
**

**G.**

**
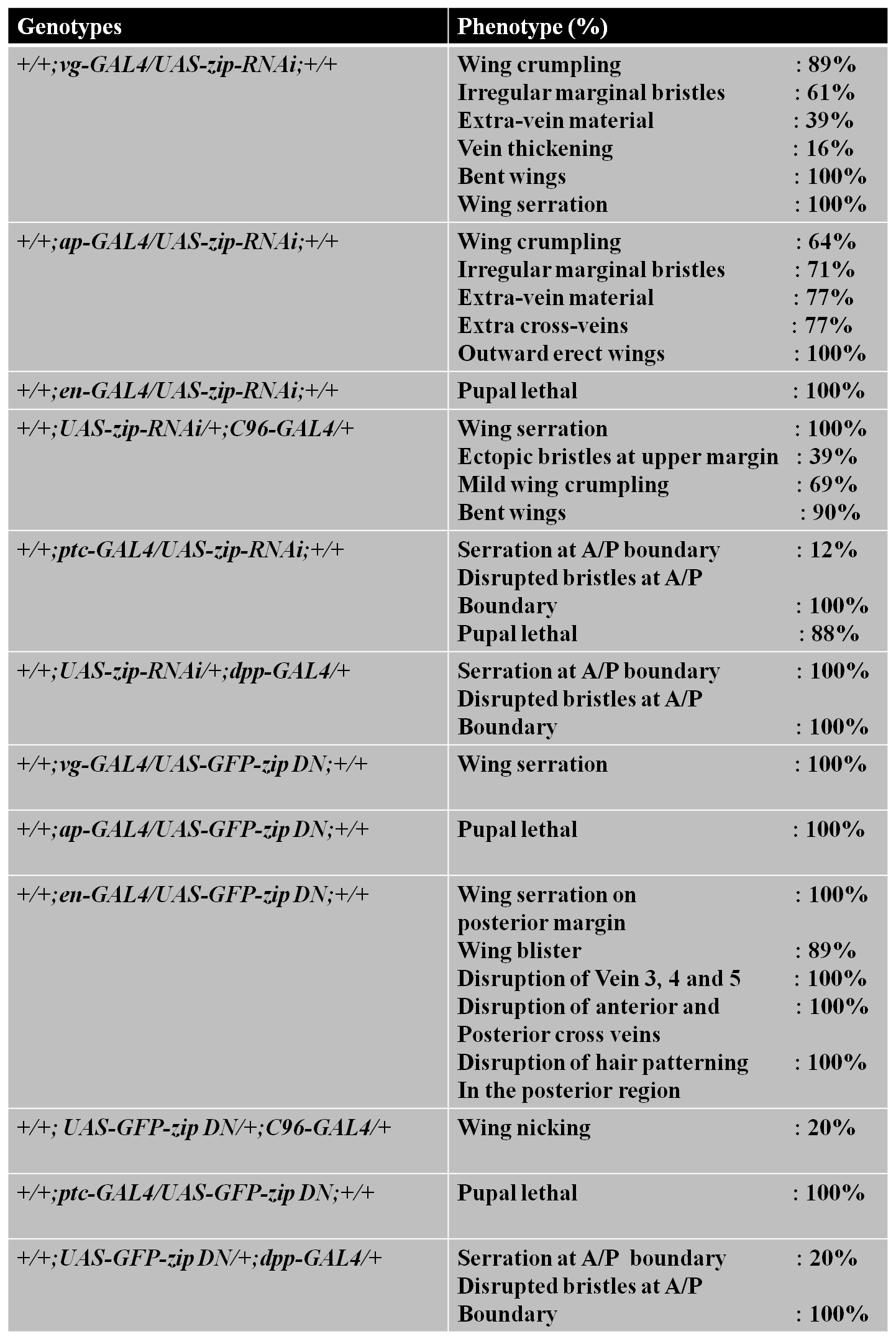
**

**Figure S2: Downregulation of *zip* results into wing phenotypes identical to Notch loss-of-function. (A-A”)** *vg-GAL4* driven *UAS-zip-RNAi* displays slightly bent wings with mild crumpling exhibiting extra-vein material, serration on the lower wing blade, irregular marginal bristles and extra cross-vein (A’) and *vg-GAL4* driven *UAS-GFP-zip DN* shows wing serration phenotype (A”) compared to the control that is almost wild-type (A). **(B-B”)** *ap-GAL4* driven *UAS-zip-RNAi* wings are directed outwards and display formation of extra cross-veins and exhibit extra vein material (B’) whereas *ap-GAL4* driven *UAS-GFP-zip DN* results in pupal lethality (B”) compared to control that is almost wild-type. **(C-C”)** *zip* upon-downregulation using *UAS-zip-RNAi* in the posterior region of the wing results in pupal lethality (C’) whereas reducing the dose of Zip using *UAS-GFP-zip DN* in the posterior region of the wing displays serration on the posterior blade of the wing margin, wing blister, disruption of the third, fourth and fifth vein, disruption of the anterior and posterior cross-veins and mispatterning of the wing hairs in the posterior region (C”) compared to the control (C). **(D-D”)** Downregulating *zip* on the marginal blade of the wing using *C96-GAL4* results into crumpled wings with serration on the lower margin of the wing blade and irregular marginal bristles (D’) whereas *C96-GAL4* driven *UAS-GFP-zip DN* results in wing nicking phenotype (D”) in comparison to control which is almost wild type (D). **(E-E”)** *ptc-GAL4* driven *UAS-zip-RNAi* results in wing nicking on the A/P boundary of the wing (E’) whereas Zip on being reduced on the A/P boundary using *UAS-GFP-zip DN* results in pupal lethality (E”). **(F-F”)** Downregulating zip at A/P boundary using *UAS-zip-RNAi* and *UAS-zip-DN* with *dpp-GAL4* leads to mild wing nicking at the wing margin of A/P boundary and disrupted wing bristles compared to the wild type control. **Scale bar: 3cm (G)** Table representing the scores of various phenotypes obtained upon downregulating *zip* with wing-specific GAL4 drivers**.**

**Figure S3:**

**
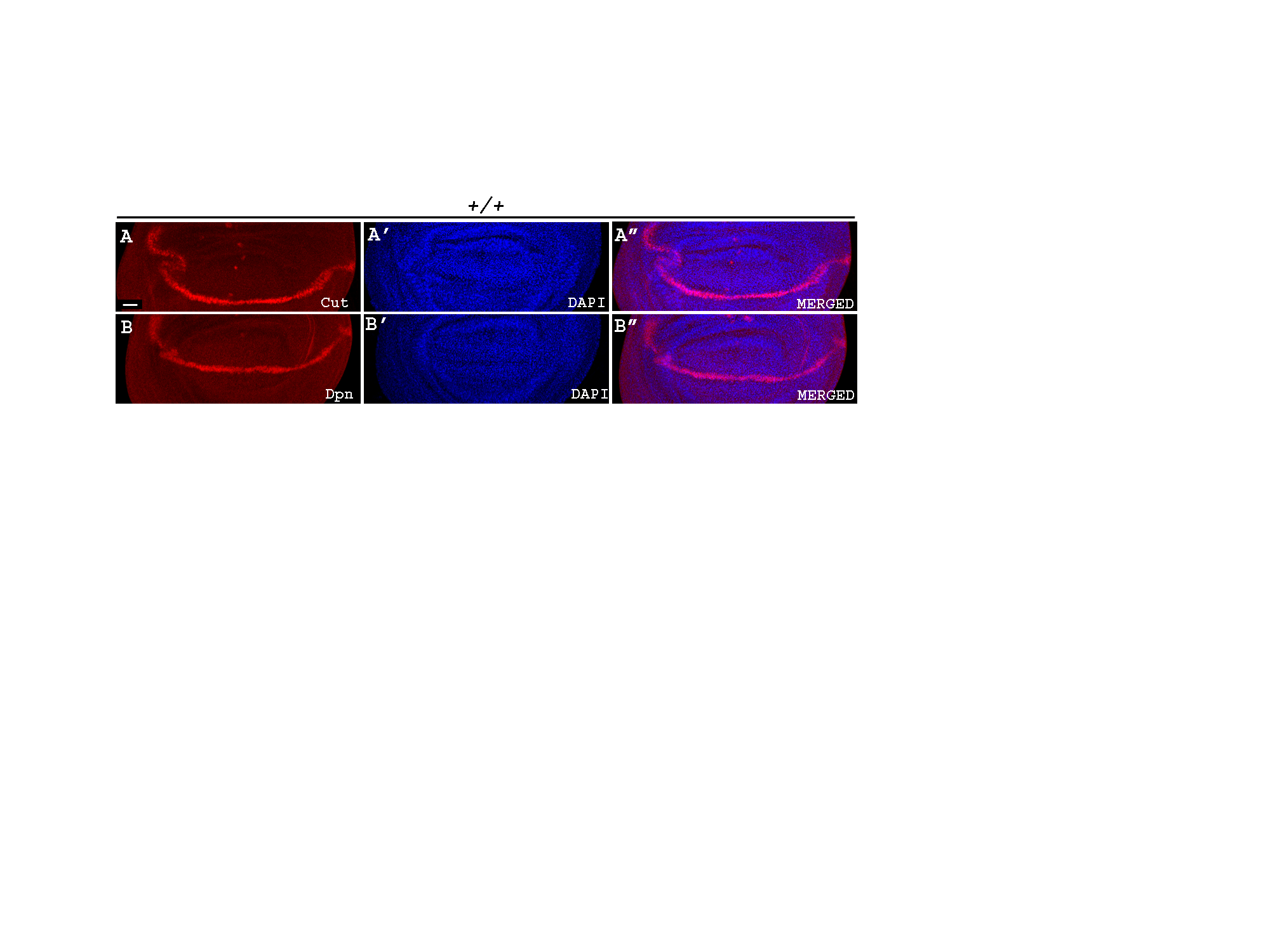
**

**Figure S3: Expression of Notch signaling targets Cut and Dpn in wild type wing imaginal discs. (A and B)** Representative wing discs depicting the Cut and Dpn expression pattern in wild type wing imaginal discs**.** Panels **A’** and **B’** shows DAPI and **A”** and **B”** shows merged images. **Scale Bar: 20µm.**

**Figure S4:**

**
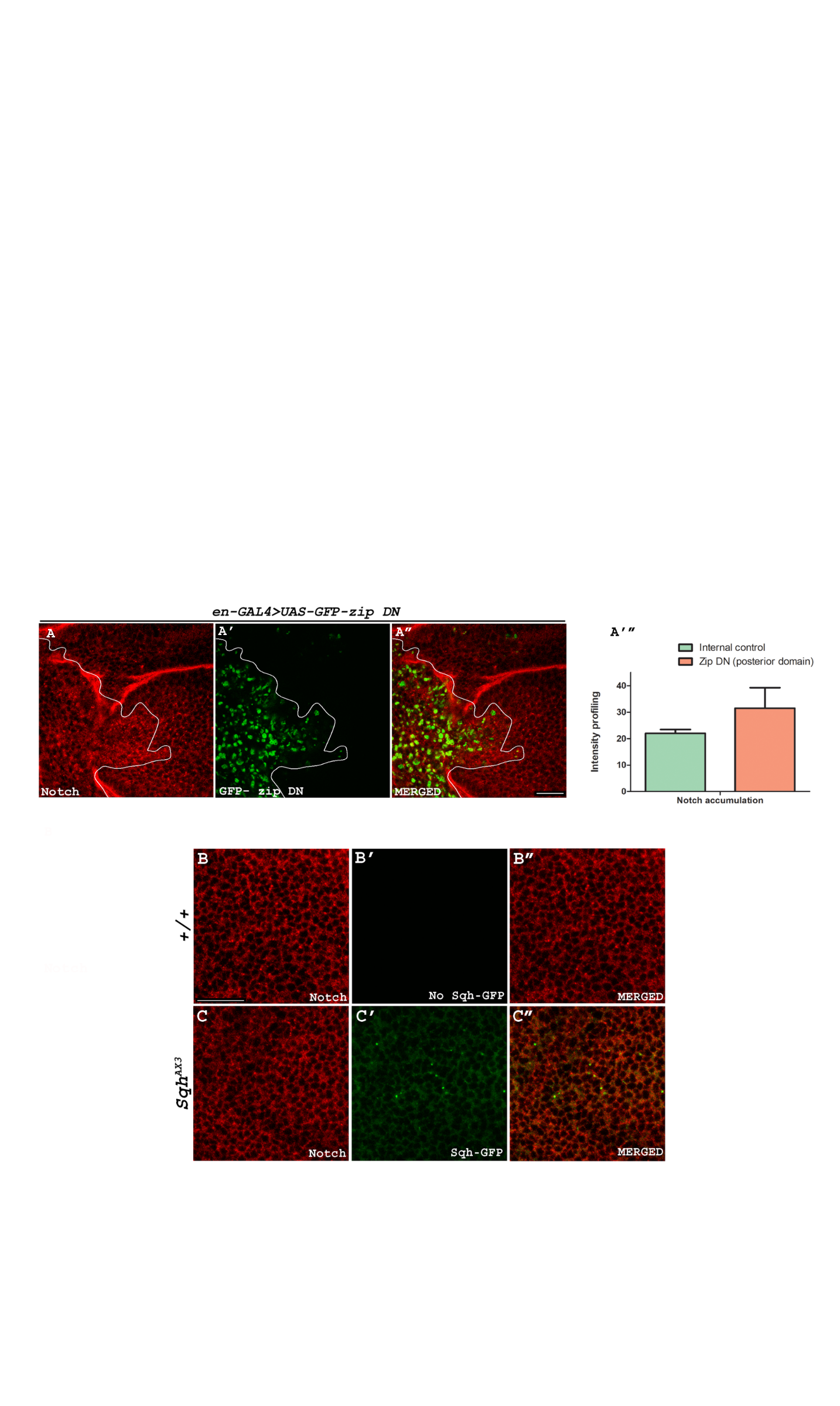
**

**Figure S4: Down regulation of Zip leads to accumulation of Notch. (A-A’’’)** Zip downregulation with *UAS-GFP-zip DN* in the posterior region of the wing disc using *en-GAL4* resulted in an accumulated expression of the Notch receptor on the cell membrane compared to its endogenous expression in the anterior domain (A). Panel A” represents a merged image of A and A”. Panel A’ shows the downregulation of Zip in the posterior domain using GFP-tagged *zip DN*. **(A’”)** Graph showing the accumulation of Notch receptor at the membrane level in the posterior region of the wing disc with compromised Zip compared to the anterior domain. **(B-C”)** Representative wing discs showing the expression of Notch in wild type (B) and null *squash* (C). Panel C’ represents the expression of GFP tagged Sqh whereas Panel C represents absence of *sqh.* Panel B” and C” represents the merged images. **Scale bar: 20µm.**

**Figure S5:**

**
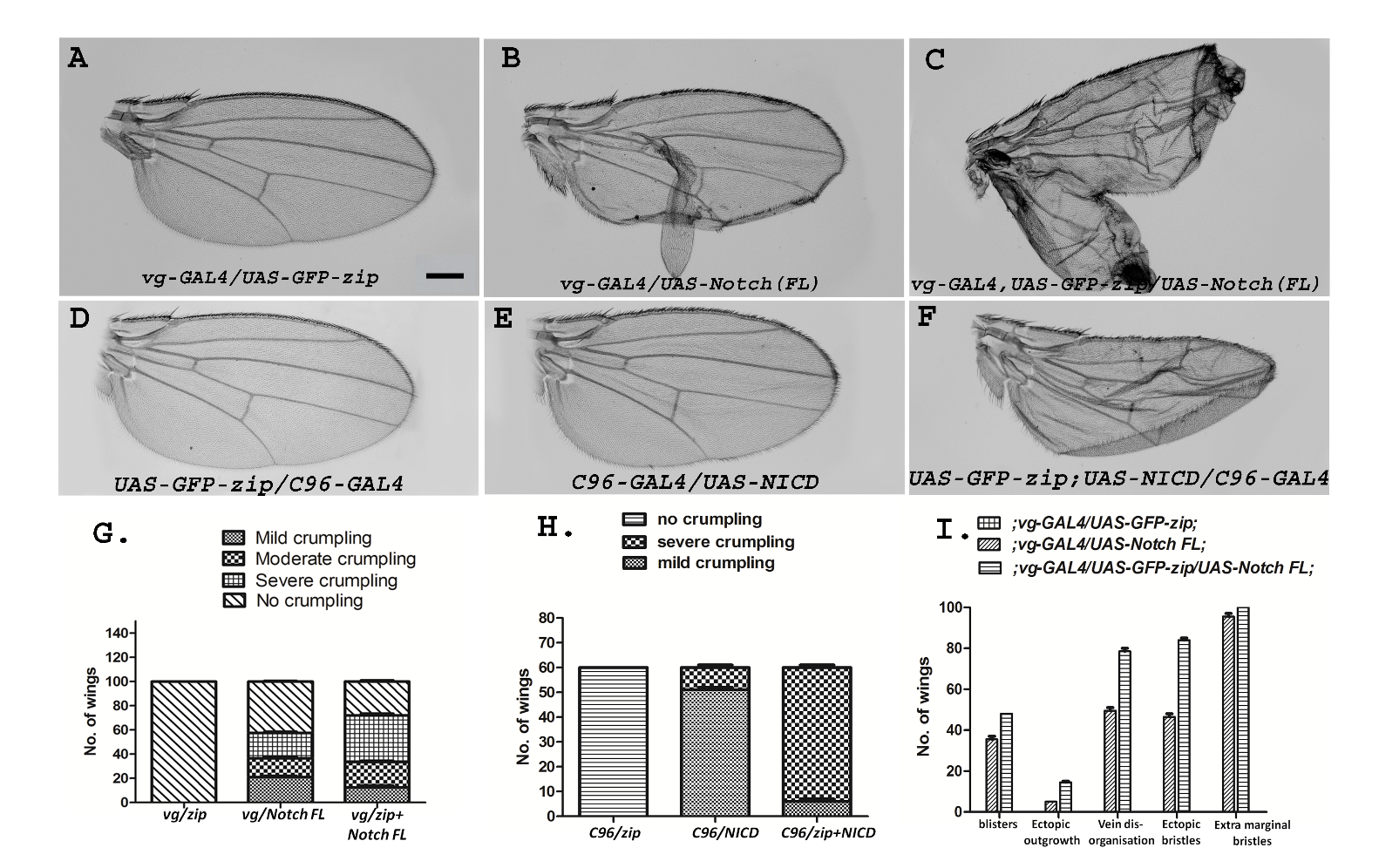
**

**Figure S5: Synergistic effect of *Notch* and *zip* results into enhanced Notch gain-of-function phenotypes**. **(A-F)** Adult wing images from the indicated genotypes. z*ip* upon over-expression by *vg-GAL4* displays wild-type wings (A). *Notch-FL* on being over-expressed under *vg-GAL4* results in wings with mild crumpling, and ectopic wing tissues (B). Panel C shows wing splitting phenotype with cup-shaped folded wings, crumpling, ectopic bristles, vein disorganisation and blisters in the adult wing upon co-expression of *Notch (FL)* and *zip* under *vg-GAL4*. **(D-E)** Similarly, *zip* synergises with processed *Notch (Notch-ICD)* under *C96-GAL4* to yield severely crumpled wing (F) compared to wild-type wing resulting upon *zip* over-expression under *C96-GAL4* (D) and mildly crumpled wings resulting due to *Notch-ICD* over-expression under *C96-GAL4* (E). **Scale bar: 3 cm** **(G-I)** Bar graphs representing the number of wings showing enhancement in wing phenotypes upon co-expression of *zip* with unprocessed (G and I) and processed (H) Notch. The genotypes mentioned on the X-axis of graph G are as follows: *vg-GAL4/UAS-GFP-zip, vg-GAL4/UAS-Notch-FL*, and *vg-GAL4/UAS-GFP-zip+UAS-Notch-FL*. The genotypes mentioned on the X-axis of graph H are as follows: *C96-GAL4/UAS-GFP-zip, C96-GAL4/UAS-Notch-ICD*, and *C96-GAL4/UAS-GFP-zip+UAS-Notch-ICD*.

**Figure S6:**

**
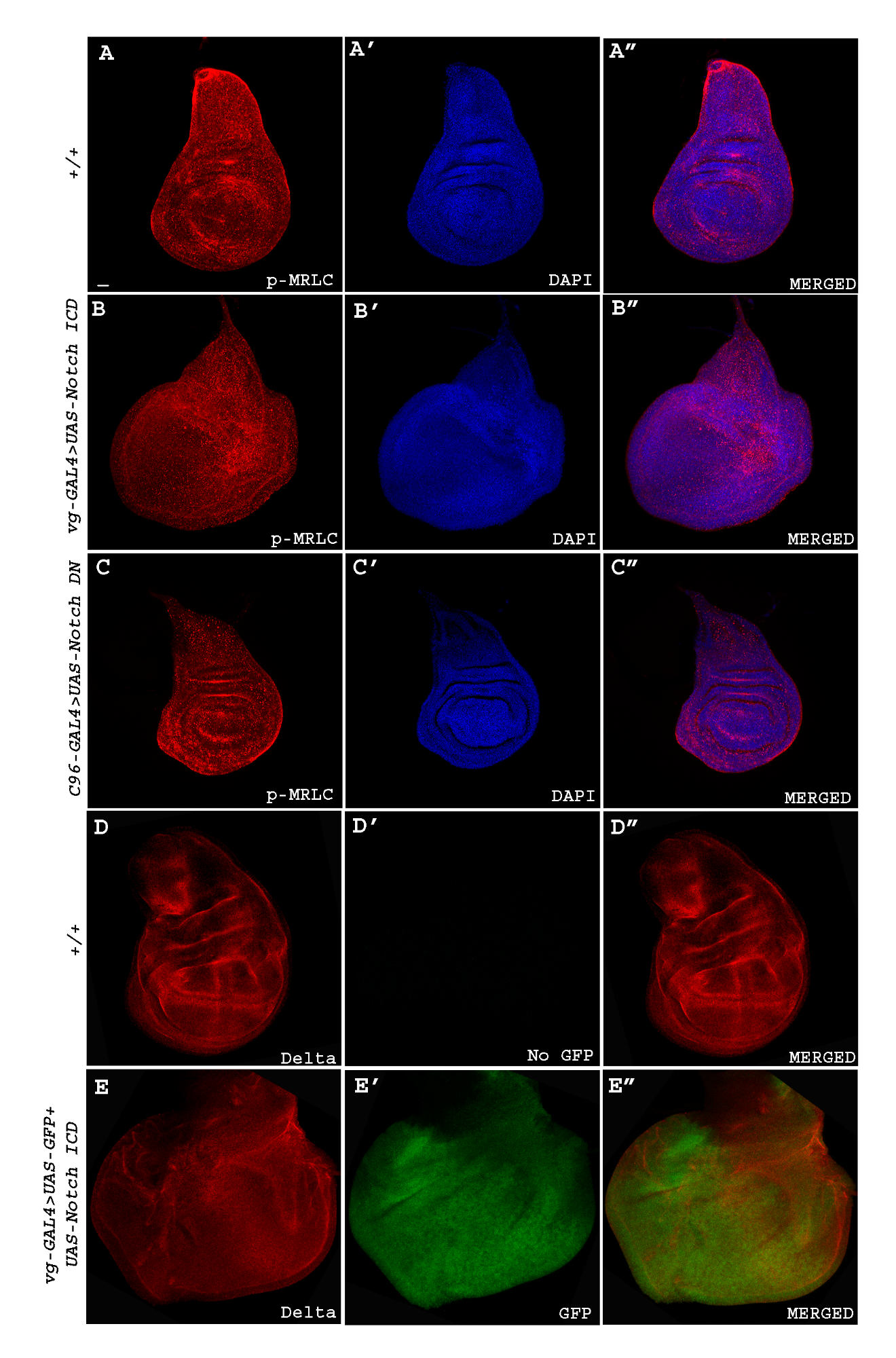
**

**Figure S6: Notch does not modulate Zip activity. (A-C”)** Representative wing discs from *Oregon R* (A), *vg-GAL4>UAS-Notch ICD* (B) and *C96-GAL4>UAS-Notch DN* (C) showing the expression of phospo-mysoin regulatory light chain. A’, B’ and C’ represents DAPI and A”,B” and C” are the merged images from the respective genotypes. **Delta expression remain altered in over-expressed Notch-ICD. (A-B”)** Representative wing discs showing the expression of Delta in wild type (A) and Notch-ICD over-expressed (B) conditions in vestigial domain. **Scale bar: 20µm**.

**Figure S7:**

**
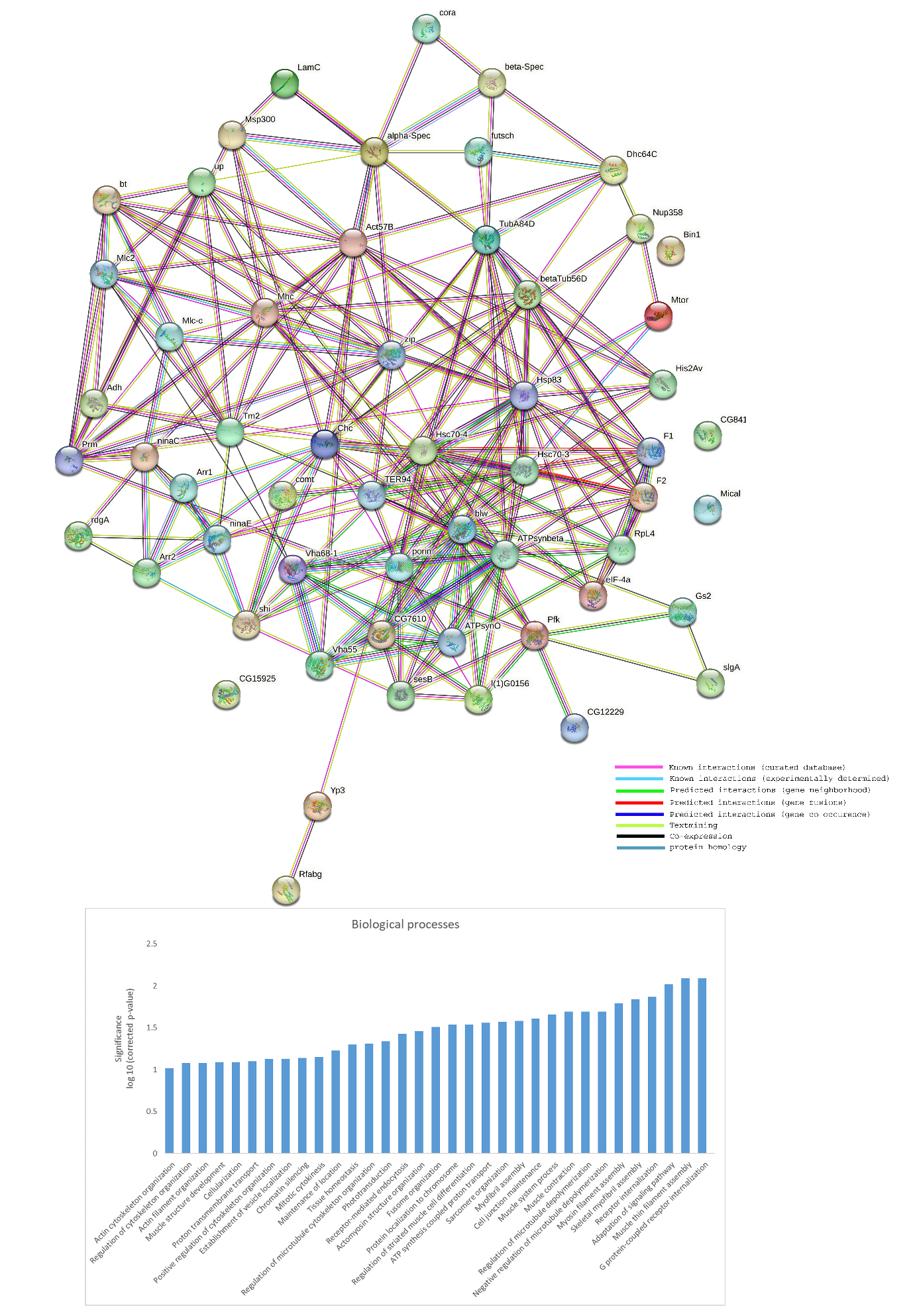
**

**Figure S7: Proteome analysis of Zip interacting partners and their involvement in biological processes.** Protein interactome showing the interacting partners of Zip identified upon co-immunoprecipitation with anti-Zip antibody followed by high resolution mass-spectrometry. The connecting lines represent known and predicted interactions amongst the different proteins. The functional enrichments of the biological processes found in this network has been shown via log 10 transformed Benjamini-Hochberg corrected p-values.
